# Antineoplastic effect of compounds C14 and P8 on TNBC and radioresistant TNBC cells by stabilizing the K-Ras4B^G13D^/PDE6δ complex

**DOI:** 10.3389/fonc.2024.1341766

**Published:** 2024-03-20

**Authors:** Dayan A. Carrión-Estrada, Arturo Aguilar-Rojas, Sara Huerta-Yepez, Mayra Montecillo-Aguado, Martiniano Bello, Arturo Rojo-Domínguez, Elena Arechaga-Ocampo, Paola Briseño-Díaz, Marco Antonio Meraz-Ríos, María del Rocío Thompson-Bonilla, Rosaura Hernández-Rivas, Miguel Vargas

**Affiliations:** ^1^ Department of Molecular Biomedicine, Center for Research and Advanced Studies of the National Polytechnic Institute (CINVESTAV-I.P.N.), Mexico City, Mexico; ^2^ Medical Research Unit in Reproductive Medicine, Mexican Social Security Institute (IMSS), High Specialty Medical Unit in Gynecology and Obstetrics No. 4 Dr. Luis Castelazo Ayala, Mexico City, Mexico; ^3^ Research Unit in Oncological Diseases, Children’s Hospital of Mexico Federico Gómez, Mexico City, Mexico; ^4^ Laboratory for the Design and Development of New Drugs and Biotechnological Innovation, Higher School of Medicine, National Polytechnic Institute, Mexico City, Mexico; ^5^ Department of Natural Sciences, Metropolitan Autonomous University Cuajimalpa Unit, Mexico City, Mexico; ^6^ Department of Biochemistry of the Faculty of Medicine of the National Autonomous University of Mexico (UNAM), Mexico City, Mexico; ^7^ Biomedical and Transnational Research, Genomic Medicine Laboratory, Hospital 1° de Octubre, Institute of Security and Social Services of State Workers (ISSSTE), Mexico City, Mexico

**Keywords:** K-Ras4B, PDE6δ, breast cancer, triple negative breast cancer (TNBC), radioresistant, antitumor compounds, C14, P8

## Abstract

**Introduction:**

Breast cancer (BC) is the leading cause of cancer-related deaths among women, with triple-negative breast cancer (TNBC) representing one of the most aggressive and treatment-resistant subtypes. In this study, we aimed to evaluate the antitumor potential of C14 and P8 molecules in both TNBC and radioresistant TNBC cells. These compounds were chosen for their ability to stabilize the complex formed by the overactivated form of K-Ras4B^G13D^ and its membrane transporter (PDE6δ).

**Methods:**

The antitumor potential of C14 and P8 was assessed using TNBC cell lines, MDA-MB-231, and the radioresistant derivative MDA-MB-231RR, both carrying the K-Ras4B> ^G13D^ mutation. We investigated the compounds' effects on K-Ras signaling pathways, cell viability, and tumor growth in vivo.

**Results:**

Western blotting analysis determined the negative impact of C14 and P8 on the activation of mutant K-Ras signaling pathways in MDA-MB-231 and MDA-MB-231RR cells. Proliferation assays demonstrated their efficacy as cytotoxic agents against K-Ras^G13D^ mutant cancer cells and in inducing apoptosis. Clonogenic assays proven their ability to inhibit TNBC and radioresistant TNBC cell clonogenicity. In In vivo studies, C14 and P8 inhibited tumor growth and reduced proliferation, angiogenesis, and cell cycle progression markers.

**Discussion:**

These findings suggest that C14 and P8 could serve as promising adjuvant treatments for TNBC, particularly for non-responders to standard therapies. By targeting overactivated K-Ras and its membrane transporter, these compounds offer potential therapeutic benefits against TNBC, including its radioresistant form. Further research and clinical trials are warranted to validate their efficacy and safety as novel TNBC treatments.

## 1 Introduction

Breast cancer (BC) is the most frequently diagnosed cancer and the leading cause of cancer deaths among women worldwide. In 2020 alone, BC accounted for 2.3 million new cases in women, representing 24.5% of the total cancer cases, with a 15.5% mortality rate among cancer cases (1, 2). This disease manifests as a highly heterogeneous and complex entity, encompassing various clinical and molecular subtypes, each posing distinct challenges. According to the expression of specific markers, such as estrogen receptor (ER), progesterone receptor (PR), epidermal growth factor receptor 2 (HER2), and the proliferation marker Ki-67, BC has been principally classified into five subtypes: luminal A, luminal B, luminal B-like, HER2-enriched, and triple-negative breast cancer (TNBC) (3). TNBC represents approximately 15%–20% of all breast cancer cases and is considered one of the most aggressive BC subtypes. It is characterized by the absence of ER, PR, and HER2 expression, coupled with elevated Ki-67 levels, leading to rapid growth, high recurrence rates, metastatic potential, worse prognosis, and limited treatment options than other BC subtypes (4, 5) (4, 6). Currently, the standard of care to treat high-risk and locally advanced TNBC is chemotherapy, radiotherapy, and, recently, immune checkpoint blockade (ICB) agents (7). Although all those therapeutic schemes have shown their effectiveness against TNBC, this is limited especially by the development of resistance, tumoral recurrences, metastasis, and the emergence of serious adverse side effects (8). For example, there is a 40% mortality rate within the first 5 years after diagnosis, and a substantial number of patients develop distant metastasis (46%) and recurrent disease after surgery (>25%) (6, 9). Additionally, the development of resistance to treatments in these patients has been observed. For example, a significant portion of TNBC patients are not able to respond to chemotherapy and radiation schemes (60%–70%). This tumoral aggressiveness has been associated with various factors. In this context, one of the most important events according to genomic landscape studies is the overactivation of KRAS and its associated signaling pathways, which are the driving force behind the malignant behavior observed in these tumors, including the acquisition of early tumor relapse, local invasion, and metastatic spread (10). Likewise, according to previous reports, patients who express overactive mutant forms of KRAS also have been associated with the development of chemoresistance (11, 12). In this case, although the development of chemoresistant phenotypes in TNBC is implicated in the activation of multiple signaling pathways, context-dependent compensatory pathway crosstalk, synergy, antagonism, and reconfiguration of signaling network (8), overactivation of the EGFR/K-RAS/MAPK pathway is highly prevalent in chemoresistant, recurrent, locally advanced, and metastatic TNBC (13).

Additionally, TNBC patients with KRAS mutations often display resistance to radiation therapy, a key component of breast cancer treatment. The resistance observed in TNBC patients may stem from the highly heterogeneous nature of these tumors such as high diversity in tumoral cell populations, the presence of cancer stem cells, and epithelial–mesenchymal transition characteristics. These factors can lead to a reduced response to radiotherapy (14, 15). Overcoming radioresistance in TNBC remains a critical area of research, with ongoing efforts focused on identifying novel therapeutic strategies to enhance the effectiveness of treatments and radiation therapy in this aggressive and challenging breast cancer subtype (15).

Considering this, KRAS has emerged as a promising therapeutic target. K-Ras4B is the more abundant isoform of the K-Ras protein (16). Mutant K-Ras4B is associated with various cancers, such as pancreatic cancer (60%), colon cancer (32%), lung cancer (17%), and approximately 5%–15% of breast cancers, including TNBC, and plays a role in tumorigenesis by driving key signaling pathways like RAF/MEK/MAPK (17). In the specific case of breast cancer, up to 23% of premenopausal women with TNBC have been shown to have higher rates of mutations in KRAS gene. Mutant KRAS in TNBC correlates with therapy resistance, reduced expression of estrogen receptor alpha (Erα), development of resistance to antiestrogen treatments, and negative prognostic outcomes, making it one of the pivotal factors in the progression of aggressive BC cases (18–20). With its role in driving TNBC, it offers an attractive target for therapeutic intervention, especially since patients with active mutant KRAS have an increased susceptibility to ovarian cancer as well (21). This not only highlights the significance of KRAS in promoting therapy resistance but also underscores its role in driving the development of the most aggressive forms of breast cancer.

Therefore, targeting the overactivated form of K-Ras4B emerges as a promising alternative for treating TNBC tumors, aiming to reduce Ras signaling-dependent pathways and signaling pathways associated with radioresistance (22). In oncogenic forms of K-Ras4B, 19 activating codon substitutions occur at codons 12, 13, or 61, displaying a specific pattern depending on the type of tumor. Notably, mutations in G12D, G12V, G12C, G13D, and Q61R collectively account for approximately 70% of all Ras-mutant patients (23). The substitution of glycine with aspartic acid at position 12 (G12D) is the most frequent, occurring in the range of 30% to 50% of solid tumors, with the highest incidence observed in pancreatic cancer and the lowest in lung adenocarcinoma but also is found in an isolated sample of TNBC (24). Another common mutation is G12V, which is the most prevalent in ovarian cancer and least common in cholangiocarcinoma. The G12C mutation is highly prevalent in lung adenocarcinoma (nearly 40%), but it occurs much less frequently in other tumors (approximately 10%) (25). G13D is primarily reported in colorectal cancer (12%) (26), but it is also found in the TNBC cell line MDA-MB-231 (27). Regarding Q61R, this codon represents only 2% of K-Ras4B mutations across all cancers and 5% in pancreatic ductal adenocarcinoma (PDAC) (28).

Although mutant forms of K-Ras4B play an essential role in the development, maintenance, and progression of breast cancer, there is limited information about the type and frequency of activating mutations in this neoplasia. For instance, the presence of the G12D mutation has been reported in an isolated sample of triple-negative breast cancer tumors (24). Similarly, the cell line MDA-MB-231 expresses the G13D form and is the only cellular model available to study the oncogenic function of K-Ras4B in BC.

Regarding the prevalence of K-Ras4B mutations in breast cancer, it has been reported to be between 7% and 12% (29). Specifically, the mutation frequency of K-Ras4B is 2% in luminal A tumors, 20% in luminal B tumors, 17.4% in HER2+ tumors, and 7.7% in TNBC (29). In this case, it is important to mention that there are few studies reporting the frequencies of mutant forms of this GTPase in breast cancer patients, and there are not enough studies demonstrating the specific types of mutations in patients with BC. As a result, the type, frequency, and effects of mutant forms of K-Ras4B on the population could be underestimated. For example, according to estimations made in 2018 by the United States, there were 268,670 new cases of breast cancer in that year. Even when considering a low frequency of K-Ras4B mutations in the population, it is estimated that there would be 3,578 cases associated with the presence of overactivated forms of K-Ras4B (30). Given the limited therapeutic options, the unfavorable prognosis, and the high amount of TNBC patients, the utilization of new drugs to decrease the mortality rate associated with this disease is essential.

However, targeting oncogenic forms of K-Ras4B has been a significant challenge due to its cellular localization requirements and the number of mutations. For this reason, our research group has been working in this area to propose a novel strategy to reduce the oncogenic potential of this protein. This new approach involves the use of a family of small molecules, including the compounds known as C14 and P8, which act as molecular staples (31–33). C14 is denoted as 2-[(3-chlorophenyl)-methyl-methyl-amino]-*N*-croman-4-yl-acetamide with a molecular weight of 344.83 g/mol. Five rotatable bonds, a hydrogen bridge donor atom, and three hydrogen bridge acceptor atoms are possessed by the molecule. The functional groups included benzopyrene or chromeno, which is a rigid structure formed by a benzene ring and a six-atom heterocycle with hydrogen at position 1 and a *N*-methylacetamide group (**Supplementary Figure 1A**). P8 is designated as 2-[4-(3-chlorophenyl)piperazin-1-yl]-*N*-[(4*R*)-chroman-4-yl]acetamide with a molecular weight of 385.9 g/mol and is an analog of the base compound C14 (**Supplementary Figure 1B**) (33). Both compounds are capable of binding to the complex formed by K-Ras4B and the phosphodiesterase subunit delta (PDE6δ) to reduce the activity of this GTPase and its associated signaling pathways. Through this strategy, it has been possible to disrupt tumor growth in xenograft mouse models, pancreatic cancer cell lines, and colon cancer cell lines as previously shown (31–33).

Considering the high mortality rate among patients with triple-negative tumors, coupled with their limited therapeutic options and the significant proportion of them developing resistance to conventional treatments such as radiotherapy, this study aims to assess the antitumor effects of the compounds known as C14 and P8 in models representing advanced stages of BC. For this purpose, the TNBC cell line MDA-MB-231 and the radioresistant TNBC MDA-MB-231RR (34) were employed. To the best of our group’s knowledge, these cell lines are the only *in vitro* models of BC that naturally express the K-Ras4B^G13D^ mutant form. For this reason, they have been selected as models to represent the most aggressive form of BC.

## 2 Materials and methods

### 2.1 *In silico* docking simulations and molecular dynamics simulations

Compounds C14 and P8 were selected through docking from the ENAMINE database 3D Diversity set (www.enamine.net). The crystallographic structure of the wild-type (K-Ras4B^WT^/PDE6δ) system was derived from PDB file 5TAR. Mutation for obtaining the K-Ras4B^G13D/^PDE6δ complex was introduced with the mutagenesis tool in Molecular Operating Environment (MOE; www.chemcomp.com) followed by local energy minimization of the new side chain and then minimization of surrounding atoms. Docking studies were performed using both MOE and GOLD (35). Protein–protein interaction energies between K-Ras4BWT/PDE6δ and K-Ras4B^G13D^/PDE6δ systems with the C14 and P8 compounds were calculated using the HawkDock Server (http://cadd.zju.edu.cn/hawkdock/), taking into account force field interactions and solvation energies (36, 37).

Extensive docking calculations were conducted on the macromolecular system, initially through a blind search of the surface, followed by a focus on the resulting main cavity. In the case of MOE, a set of conformers was prepared using the MMFF94x force field, followed by five rounds of extensive testing; 15,000 poses for each conformer on the receptor target (crystallographic interface between K-Ras4B with PDE6δ) were evaluated. For GOLD, 100 different runs of the Genetic Algorithm were performed and scored using both ChemPLP and Goldscore. The best results from each program were rescored using the other software to compare the outcomes. The best overall score for each pose was selected for subsequent molecular dynamics simulations.

The molecular dynamics (MD) simulations of the wild-type and mutated K-Ras4BWT/PDE6δ–ligand complexes in the presence of C14 and P8 poses with the best binding scores predicted through docking studies were performed using MOE. For the K-Ras4BWT/PDE6δ–ligand complex, a periodic rectangular-shaped box of 48.7 × 73.1 × 60.9 Å was used with TIP3P water model (38–41). Cl^−^ and Na^+^ ions for the protein–ligand system were placed in the model to neutralize the positive or negative charges around the complex at pH 7. Before the MD simulation, the system was minimized through 3,000 steps of steepest descent minimization, followed by 3,000 steps of conjugate gradient minimization. Then, the systems were heated from 0 K to 310 K during 500 picoseconds (ps) of MD with position restraints under an NVT ensemble. Subsequent isothermal, isobaric ensemble (NPT) of MD was carried out for 500 ps to adjust the solvent density followed by 600 ps of constant pressure equilibration at 310 K using the SHAKE algorithm (42) on hydrogen atoms, Langevin dynamics for temperature control, and a 12-Å cutoff for Van der Waals interactions. The equilibration run was followed by 100-ns-long MD simulations without position restraints under periodic boundary conditions using an NPT ensemble at 310 K. The particle mesh Ewald method was utilized to describe the electrostatic term (43). Temperature and pressure were preserved using the weak coupling algorithm (44) with coupling constants τT and τP of 1.0 ps and 0.2 ps, respectively (310 K, 1 atm). The time of the MD simulation was set to 2.0 femtoseconds, and the SHAKE algorithm (42) was used to constrain bond lengths at their equilibrium values. Coordinates were saved for analyses every 50 ps. AmberTools14 was used to examine the MD runs and clustering analysis to identify the most populated conformations during the equilibrated simulation time.

### 2.2 Calculation of binding free energies

Binding free energies were calculated using the molecular mechanics with a generalized Born and surface area (MMGBSA) approach (45–47) provided in the AMber16 suite (40). A total of 500 snapshots were chosen at time intervals of 100 ps from the last 50 ns of MD simulation using a concentration of 0.1 M and the generalized Born (GB) implicit solvent model (48). The binding free energy of the protein–ligand system was determined as follows:


(1) $$\Delta {\text{G}}_{\text{bind}}=\Delta {\text{G}}_{\text{system}}-\Delta {\text{G}}_{\text{receptor}}-\Delta {\text{G}}_{\text{ligand}}$$

(2) $$\Delta {\text{G}}_{\text{bind}}=\Delta {\text{E}}_{\text{forcedield}}+\Delta {\text{G}}_{\text{solvation}}-\text{T}\Delta \text{S}$$

ΔE_forcefield_ represents the molecular mechanical force field’s total energy, including the electrostatic (ΔEele) and van der Waals (ΔEvdw) interaction energies. ΔG_solvation_ is the free desolvation energy price upon complex formation estimated from the GB implicit model and solvent-accessible surface area (SASA) calculations yielding ΔGele,sol, and ΔGnpol,sol. TΔS is the solute entropy arising from structural changes in the free solutes’ degrees of freedom when forming the protein–ligand complex.

Binding free energies along with their constituent energy components are presented for the complexes based on initially docked conformations, expressed in kcal/mol. The breakdown includes polar contributions (ΔEpolar = ΔEele + ΔGele,sol) and non-polar contributions (ΔEnon-polar = ΔEvwd + ΔGnpol,sol). All energy values have been averaged over 500 snapshots, obtained at 100-ps intervals during the concluding 50 ns of the MD simulations. The average standard error in ΔG_bind_ amounts to 9 kcal/mol.

### 2.3 Cell culture

The human mammary cancer cell line MDA-MB-231 [HTB-26, American Type Culture Collection (ATCC) (Manassas, VA, USA)] was employed as TNBC cells. In this case, this cell line was selected.

MCF-7 [HTB-22, ATCC] as luminal A model and MCF-10A [CRL-10317, ATCC] as non-tumoral cells. All of them were purchased from the ATCC (Manassas, VA, USA). MDA-MB-231 cells were cultured in Leibovitz’s medium supplemented with antibiotics and 10% fetal calf serum (FCS) (Invitrogen, Carlsbad, CA, USA). MCF-7 was cultured in Dulbecco’s modified Eagle’s medium (DMEM) (Invitrogen) also supplemented with 10% FCS and a mix of antibiotics. MCF-10A was cultivated in DMEM/F12 (Invitrogen) cell media, supplemented with 10% fetal bovine serum (FBS) and a mix of antibiotics. Finally, as radioresistant TNBC cells, the cell line MDA-MB-231RR was employed, which was kindly donated by Professor Elena Arechaga-Ocampo and grown as previously reported (34). All the cell lines were maintained under growth conditions at 37°C and 5% CO_2_ in a humidified atmosphere and used between 5 and 10 passage numbers.

### 2.4 Microscopy assays

Structural characteristics of the cellular models employed were evaluated by confocal microscopy of the F-actin arrangement following the procedure described elsewhere (49). In brief, cells were cultured on coverslips for 24 h. Then, F-actin was stained with phalloidin-rhodamine (Invitrogen) as described above, mounted on slides, and covered with Vectashield as an antifade mounting medium (Invitrogen). All the samples were then visualized in an A1 confocal microscope (NIKON, Tokyo, Japan) at 580-nm excitation and 604-nm emission (50).

### 2.5 Sequencing of exon 2 of *KRAS* gene

To verify the presence of the mutation c.38G>A in the exon number 2 of *kras* gene, genomic DNA (gDNA) was extracted from MCF-10A, MDA-MB-231, and MDA-MB-231RR cell lines and sequenced. gDNA was purified using the GenElute Mammalian Genomic DNA (gDNA) miniprep kit (Sigma-Aldrich, St. Louis, MO, USA). Subsequently, quantification of each gDNA sample was performed using NanoDrop 2000 equipment (Thermo Fisher Scientific, Waltham, MA, USA). Then, the integrity of each genomic DNA sample was verified by agarose gel electrophoresis. Both strands of the exon 2 of *kras* gene were sequenced with approximately 60 ng of gDNA as a template and the BigDye Terminator v3.1 Cycle Sequencing Kit (Thermo Fisher Scientific) according to the provider’s instructions. The following specific oligonucleotides (10 pM/µl) were employed for this purpose (NCBI Reference Sequence: NM_004985.5):

Forward: RASO1 5′-AAGGCCTGCTGAAAATGAC-3′,

Reverse: RASA2 5′-TGGTCCTGCACCAGTAATATG-3.

Electropherograms obtained were verified using the software ChromasPro 1.7.7 (51).

### 2.6 Wound-healing migration assay

To determine the cell migration ability of MCF-10A, MDA-MB-231, and MDA-MB-231RR cell lines, wound-healing migration assays were performed. In brief, every cell line was seeded into 6-well culture plates (Corning, New York, NY, USA) and cultured in a medium containing 10% FBS until confluent. Then, a wound was made on the cell monolayer by scratching it with a sterile 200-µl micropipette tip. Any cellular debris that was present was removed by washing with phosphate-buffered saline (PBS). Cells were allowed to migrate at 37°C in 5% CO_2_. Images of the wounded areas were taken at 0, 2, 4, and 6 h using a Leica epifluorescence microscope, with a 10× objective (Leica, Wetzlar, Germany). All experiments were performed in triplicate incubations. The images were analyzed with the aid of Leica software (Leica) (52).

### 2.7 Invasion assay

The invasion ability of MCF-10A, MDA-MB-231, and MDA-MB-231RR cells was evaluated by invasion of Transwell chambers coated with Basement Membrane Matrix Growth Factor Reduce Matrigel (Corning). MCF-10A, MDA-MB-231, and MDA-MB-231RR cells were seeded in the upper chamber of the Transwell at 250,000 cells/100 μl in serum-free media. In the lower chamber, 10% FBS was added as a chemoattractant. As a control, chambers without FCS were employed. Under all conditions, the cells were allowed to migrate to the lower chamber for 24 h at 37°C in 5% CO_2_ (53). Each condition was performed in triplicate. After this time of incubation, the non-migrated cells on the upper side of the porous membrane were removed using a cotton swab soaked with PBS. The cells that migrated across the porous membrane were fixed using 4% paraformaldehyde (PFA) and then stained with 0.1% Giemsa stain for cell counting using a Leica epifluorescence microscope with 10× objective (Leica). Afterward, the dye retained in the insert was extracted and transferred to a 96-well microtiter plate to be measured at 560 nm (54).

### 2.8 Quantitative pseudopodia assay

The number of cellular extensions present in MCF-10A, MDA-MB-231, and MDA-MB-231-RR cells was studied using the Chemicon Quantitative Pseudopodia Assay Kit (Corning). This system allows the insolation and quantification of extending or retracting pseudopodia from the cell body. In brief, porous membranes of Pseudopodia Quantification Inserts were coated with a Basement Membrane Matrix Growth Factor Reduce Matrigel (Corning). Later, the coated plates were incubated for 2 h to allow the gel to polymerize. Then, in the upper chamber of the inserts, 250,000 cells/100 μl of MCF-10A, MDA-MB-231, and MDA-MB-231RR were seeded in serum-free media. In the lower chamber of the inserts, 10% FBS was added as a chemoattractant. As a negative control, cells without chemoattractant were employed. All the samples were maintained for 2 h of incubation to leave an extension of pseudopodia through the pores of the membrane. At the end of the time, every insert was rinsed twice with 1× PBS, and the cell body was removed from the upper membrane surface by wiping with cotton. The cells that extend pseudopodia across the porous membrane were fixed with 4% PFA and then stained with Pseudopodia Stain Solution (Corning). Then, each insert was rinsed with water, and stained Pseudopodia was eluted with Stain Extraction Buffer (Corning). Eluted samples were measured at O.D. 600 nm in a microplate Synergy-HTX microplate reader (BioTek, Winooski, VT, USA). Each condition was performed in triplicate (55).

### 2.9 Cell viability assay and IC50 determination

In order to evaluate the effect of the C14 and P8 compounds over MCF-10A, MCF-7, MDA-MB-231, and MCF-MB-231-RR cell lines, the IC50 of those molecules was determined. In brief, all the cell lines were seeded at a density of 15,000 cells per well in a 96-well microtiter plate (Corning) in a corresponding growth medium for 24 h. Then, cells were treated with increasing concentrations (from 0 to 200 μM) of the C14 and P8 compounds (Enamine, Kyiv, Ukraine). As a positive control, increased concentrations of cisplatin (from 0 to 200 µM) (Accord, Mexico City, Mexico) were employed. Due to that, dimethyl sulfoxide (DMSO) was used as a vehicle, and its increased concentrations were evaluated as the negative control. All the samples were maintained under growth conditions for 24 h and 48 h. At the end of each time, cell viability was determined using the XTT Cell Proliferation Kit II (Roche Applied Science, Mannheim, Germany) following the manufacturer’s protocol. The absorbance was measured in a spectrophotometer Synergy-HTX (BioTek) at a wavelength of 450–500 nm with a reference wavelength of 650 nm. Cell proliferation was expressed as a percentage of viability [(absorbance of treated cells/absorbance of untreated cells × 100)] ± SD. All the assays were performed in triplicate, and IC50 values for 24 h (IC50-24) and 48 h (IC50-48) of each sample were calculated using the Prism 8 software (GraphPad, La Jolla, CA, USA).

### 2.10 Apoptosis assay

To determine if the C14 and P8 compounds were able to evoke cell death by apoptosis or necrosis, cell lines MCF-10A, MDA-MB-231, and MDA-MB-231RR were treated at IC50-24 for 24 h. Apoptosis and necrosis were determined using the Apoptosis/Necrosis Detection kit (Abcam, Cambridge, UK) according to the manufacturer’s instructions. In brief, approximately 500,000 cells were seeded in 6-well plates (Corning) for 24 h. Then, each cell line was treated with the respective IC50-24 concentrations of C14 and P8. As positive controls, 100 µM cisplatin and 0.5 µM doxorubicin were used as the compounds for 24 h. Cells were harvested and collected by centrifugation. All the samples were analyzed in triplicate using flow cytometry equipment FACSCalibur instrument (BD Biosciences, San Jose, CA, USA) at 530-nm excitation and 575-nm emission. Data analysis was performed using the FlowJo software (Tree Star Inc., Ashland, OR, USA). All experiments were performed in triplicate.

### 2.11 Clonogenic assay

The principal aim of a clonogenic assay is to evaluate the effect of chemotherapy agents or new drugs by the measurement of their ability to arrest tumor cell division and their ability to develop new colonies after their exposure to these new drugs (56). Therefore, the colony formation ability of the cell lines MCF-10A, MDA-MB-231, and MDA-MB-231RR was evaluated after their exposure to the C14 and P8 compounds. In brief, breast cancer cell lines were cultured in 6-well plates (Corning), seeding 300 cells per well, and incubated at 37°C in 5% CO_2_ for 24 h. Compounds C14 and P8 were added at the maximum evaluated concentration IC50-48. As controls, cells without treatment and cells treated in the presence of a vehicle (DMSO at 0.66%) were employed (Sigma-Aldrich). As a positive control, cells treated with conventional chemotherapeutic agents cisplatin (100 µM) and doxorubicin (0.5 µM) were employed. After 10 days of treatment, cells were fixed with 4% PFA and stained with 0.1% crystal violet in citric acid (Sigma-Aldrich) for 15 minutes. Subsequently, the dye present in the cells was extracted using isopropanol and read at 570 nm in a Synergy-HTX microplate reader (BioTek). Each test was performed in triplicate. Data were expressed as % colonies relative to the untreated control (56).

### 2.12 Ras activation assay

The inactivation of Ras by the C14 and P8 compounds, cisplatin, and doxorubicin was determined by pull-down assays using a Ras activation assay Biochem kit (Cytoskeleton, Denver, CO, USA). The cells were serum-starved for 16 h and pre-treated with C14 and P8 at IC50-48H concentration for 3 h or cisplatin and doxorubicin for 3 h. Subsequently, the cells were stimulated with epidermal growth factor (EGF) (100 ng/ml) for 10 minutes. Lysates (1 mg/ml) were exposed to Ras GTP-binding protein (Raf-RBD), following the manufacturer’s instructions. Experiments for each cell type and condition were repeated three times.

### 2.13 Western blotting assays

To determine the effect of the C14 and P8 compounds over the protein effectors of K-Ras4B, AKT, and ERK, immunoblotting assays were conducted with specific antibodies. In brief, MCF-10A, MDA-MB-231, and MDA-MB-231RR cells were plated in 100-mm culture dishes until 80% of confluents. Cell adhesion was allowed for 24 h. Later, cells were serum-starved for 16 h to be sequentially pre-treated with the IC50-48 of C14 and P8 for 3 h. Additionally, cells were exposed to cisplatin (100 µM) and doxorubicin (0.5 µM) for 3 h. After pre-treatment, cells were stimulated with 100 ng/ml EGF for 10 minutes to promote AKT and ERK activation. Later, the whole-cell extracts were obtained by the lysis of the cells (Cytoskeleton) in the presence of proteases and phosphatase inhibitors. Subsequently, the protein extracts were incubated at 4°C for 40 minutes to be clarified by centrifugation for 10 minutes at 14,000 rpm, at 4°C. The protein concentration of each extract was determined by using the Precision Red Advanced Protein Assay Reagent (Cytoskeleton) Then, approximately 25 μg of protein extract was electrophoresed in 10% sodium dodecyl sulfate–polyacrylamide gel electrophoresis (SDS-PAGE) and transferred to polyvinylidene fluoride (PVDF) membranes (Millipore, Billerica, MA, USA). Blots were probed using the following primary antibodies: Total ERK (Cell Signaling, Danvers, MA, USA), pERK (Cell Signaling), Total AKT (Cell Signaling), pAKT (Cell Signaling), GSK3β (Cell Signaling), and cyclin D1 (Abcam) at a 1:1,000 dilution. As a control, an anti-GAPDH antibody (GeneTex, Irvine, CA, USA) was employed at a 1:100,000 dilution and γ-Tubulin (Invitrogen) 1:5,000. Densitometric analysis of blots was performed using the software ImageJ version 1.45 (National Institutes of Health, Bethesda, MA, USA).

### 2.14 Cell cycle analysis

MCF-10A, MDA-MB-231, and MDA-MB-231RR cells (1 × 10^6^ cells/well) were either untreated (control group) or treated with C14, P8, cisplatin, and doxorubicin at IC50-48H dose for 3 h. After 3-h incubation, cells were harvested, washed twice in ice-cold PBS, and fixed overnight in 70% ethanol at 4°C. Then, cells were washed in PBS, collected by centrifugation, and stained with staining buffer (PBS with 50 µg/ml propidium iodide and 100 µg/ml RNase A).

### 2.15 Treatment of orthotopic breast carcinoma xenografts

Female immune-deficient Nu/Nu nude mice at 6–8 weeks of age (CINVESTAV, Mexico City, Mexico) were maintained in pathogen-free conditions with irradiated chow. The animals were subcutaneously injected in the left 4th mammary gland with 2 × 106 MDA-MB-231RR cells per tumor in 0.1 ml of sterile phosphate-buffered saline. When MDA-MB-231RR cells reached palpable tumors (≥100 mm^3^), mice were divided randomly into four groups receiving vehicle (10% DMSO, 0.05% carboxy methyl cellulose, and 0.02% in PBS) (n = 5), C14 at 30 mg/kg (n = 5), P8 at 10 mg/kg (n = 5), or cisplatin at 6 mg/kg (n = 5) intraperitoneally injected daily for 15 days. Body weight and tumor volume were measured every third day. Tumor sizes were calculated using the formula [(length × width2)/2] in mm.

### 2.16 Immunohistochemistry assays and digital pathology analysis

To determine the impact of C14 and P8 drugs on tumor inhibition, angiogenic, and cell cycle markers in a xenograft model, immunohistochemical assays and digital pathological analysis were conducted. For tissue preparation, 4-µm-thick tumor sections underwent a series of preparations: i) deparaffinization in xylene, ii) antigen retrieval in a sodium citrate buffer at pH 6, iii) blocking of endogenous peroxidase activity using a 10% hydrogen peroxide solution, and iv) non-specific binding blockade for 1 h. For the antibody incubation, the tumor sections were incubated with primary antibodies: Anti-Cyclin D1 (Abcam), Anti-PCNA (Abcam), Anti-CD31 (Abcam), Anti-VEGF (Abcam) at a 1:500 dilution.

The incubation with primary antibodies occurred at room temperature overnight. Subsequently, secondary antibody incubation was conducted. Then, the sections were incubated with horseradish peroxidase (HRP)-conjugated secondary antibody for 30 minutes. Finally, visualization and staining were performed using a diaminobenzidine (DAB) detection system from Vector Laboratories, Inc. (Burlingame, CA, USA). Counterstaining was performed using hematoxylin.

For the mitotic index evaluation, hematoxylin and eosin (H&E) staining was conducted to calculate the mitotic index. From each H&E-stained tumor tissue, 10 random fields were captured at ×40 magnification, and the number of mitotic figures was counted. The average count was used to calculate the mitotic index using the following formula: Mitotic index = Number of mitoses/10.

For digital pathology analysis, the immunohistochemistry (IHC)-stained sections were digitized using an Aperio ScanScope CS2 from Leica Biosystems (Nussloch, Germany), which generated high-resolution ×20 digital images (0.45 µm/pixel). These images were analyzed using ImageScope (Aperio, San Diego, CA, USA) to quantify marker expression. Then, a quantification algorithm was developed for each tissue to assess total and nuclear protein expression. The ImageScope allowed setting thresholds for color saturation and defining upper and lower limits for intensities of weak, moderate, and strong positive pixels. Lastly, the raw data encompassed the number of positive pixels and the intensity of positive pixels, which were normalized to the number of total pixels counted in µm^2^. Data were presented as total density per µm^2^.

### 2.17 Statistical analysis

All data were analyzed using Prism 8 software (GraphPad). Likewise, all of them were expressed as the mean ± standard deviation (SD). Experimental points were gathered for a minimum of three independent experiments. An unpaired Student’s t-test was used for the comparison of two groups. A value of *p*< 0.05 was considered significant.

## 3 Results

### 3.1 P8 stabilizes the K-Ras4B^G13D^/PDE6δ system more effectively than C14

As previously shown by our research group, the C14 and P8 compounds belong to the family of molecules that are able to specifically bind to and stabilize the mutated complex of K-Ras4B^mut^/PDE6δ in G12C, G12V, and G12D K-Ras4B mutant pancreatic cell lines, disrupting its localization, activation, and inhibition of oncogenic Ras signaling in pancreatic cancer cells (33). For this reason, we evaluated if these compounds were also capable of stabilizing the complex formed by the exclusive mutant form of K-Ras4B reported in breast cancer K-Ras4B^G13D^ and this membrane transporter PDE6δ (K-Ras4B^G13D^/PDE6δ) to inhibit its oncogenic activity.

**Figure 1:**
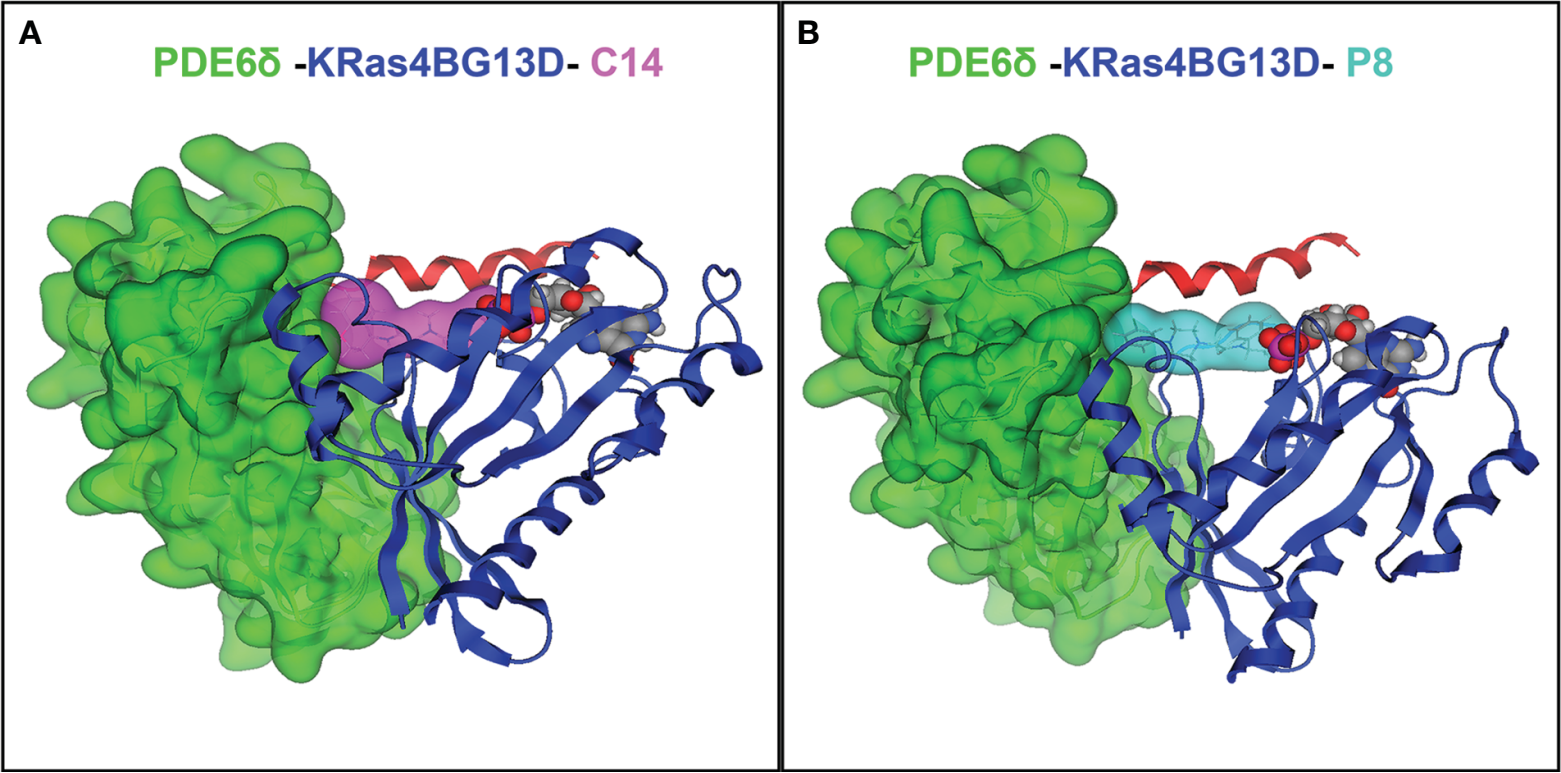
Protein–ligand interactions between compounds C14 and P8 and the K-Ras4B^G13D^–PDE6δ-HVR2 system. **(A)** Complex of K-Ras4B^G13D^/PDE6δ-HVR2 and C14. **(B)** Complex of K-Ras4B^G13D^/PDE6δ-HVR2 and P8. The interaction energy between PDE6δ protein and the GTPase K-Ras4B^G13D^ in the complex increases due to the action of compounds C14 and P8. K-Ras4B^G13D^ is shown in blue, PDE6δ-HVR2 protein in green, C14 in purple, and P8 in cyan. Position of GDP nucleotide in the complex (spheres in red, gray, and purple) is observed in the images.

To evaluate the ability of C14 and P8 to stabilize the mutated complex K-Ras4B^G13D^/PDE6δ, docking and MD simulations were performed. The lowest binding free energy poses of C14 and P8 within the K-Ras4B^G13D^–PDE6δ-HVR2 system, which were predicted through docking studies, were used as starting conformers to run 100-ns-long MD simulations. Representative protein-ligand conformations were obtained over the equilibrated simulation time (last 50 ns) using clustering analysis. Structural analysis of the representative conformations showed that C14 within the K-Ras4B^G13D^–PDE6δ-HVR2 system was bound through hydrophobic interactions by five residues from K-Ras4B^G13D^ (R41, I36, Y64, Y40, and M67) and PDE6δ (F96, F94, F92, L108, and F91) (**Figure 1A**; **Supplementary Table 1**). P8 within the K-Ras4B^G13D^–PDE6δ-HVR2 complex was stabilized through non-polar interactions by the same five residues of K-Ras4B^G13D^ (**Supplementary Table 1**) observed for the C14 compound and five residues of PDE6δ (F96, F94, F92, L108, and Q106) (**Figure 1B**; **Supplementary Table 1**). These results show that both compounds interact with the same amino acid residues, which suggests that the simultaneous binding of the two compounds could indicate a competitive interaction with the K-Ras4B^G13D^ mutant complex. This competition for the binding site was also verified by synergic assay where the cytotoxic effect of both compounds was almost additive but not synergic.

Based on free energy (ΔGbind) data calculated using Equations 1 and 2, for free K-Ras4B^G13D^/PDE6δ-HVR2, this energy was increased in complexes that contain C14 or P8. **Table 1** shows that all the complexes exhibited favorable ΔGbind values, where the non-polar (ΔEnon-polar = ΔEvwd + ΔGnpol,sol) contributions guided the molecular recognition. The ΔGbind values also showed that K-Ras4B^G13D^–PDE6δ binding was energetically more favorable for P8 (−497.8 kcal/mol) than for C14 (−490.2 kcal/mol). These results showed that the mutant form of KRas4B, KRas4B^G13D^, improved its affinity for PDE6δ compared with the wild-type system from −406.9 to −484.9 kcal/mol. KRas4B^G13D^ also increased its affinity for PDE6δ when C14 or P8 stabilized the K-Ras4B^G13D^/PDE6δ system compared with free K-Ras4B^G13D^/PDE6δ or K-Ras4B^WT^/PDE6δ systems (**Table 1**). Comparing compound C14 to P8 revealed that compound P8 may be better capable of stabilizing the K-Ras4B^G13D^/PDE6δ system.

**Table 1:** Binding free energy components of protein–protein complexes (in kcal/mol units).

System	ΔE_non-polar_	ΔE_polar_	ΔG_bind_
Protein–protein free and bound wild-type and mutated K-Ras4B^G13D^–PDE6δ			
K-Ras4B^WT^/PDE6δ	−80.2	−326.6	**−406.9**
K-Ras4B^G13D^/PDE6δ	−79.1	−405.8	**−484.9**
K-Ras4B^G13D^/PDE6δ-C14	−78.8	−411.4	**−490.2**
K-Ras4B^G13D^/PDE6δ-P8	−60.9	−437.0	**−497.8**

Binding free energies and individual energy terms of complexes starting from docked conformations (kcal/mol). The polar (ΔEpolar = ΔEele + ΔGele,sol) and non-polar (ΔEnon-polar = ΔEvwd + ΔGnpol,sol) contributions are shown. All the energies are averaged over 500 snapshots at time intervals of 100 ps from the last 50-ns-long molecular dynamics (MD) simulations and are in kcal/mol ( ± standard error of the mean).

The bold values signify Gibbs free energy of interaction between compounds and target proteins, indicating spontaneous interaction efficiency. Negative values indicate an energetically favorable interaction suggesting thermodynamically favorable binding, likely to occur spontaneously under given conditions.

### 3.2 C14 and P8 compounds decrease cellular viability of TNBC cell lines

According to these results, C14 and P8 may have a cytotoxic effect on breast cancer cells. To validate this hypothesis, we evaluated the effect of both molecules in the cell lines MCF-10A as a control and MDA-MB-231 as TNBC cells that naturally express the mutant form K-Ras4B^G13D^. TNBC K-Ras mutant has a high propensity for developing resistance to radiation therapy. This resistance often leads to increased cancer recurrence and more aggressive behavior. By using a radioresistant cell line MDA-MB-231RR, we simulated the clinical challenges of treating radioresistant breast cancer, which is crucial for assessing the effectiveness of potential therapeutic agents. We verified the presence of K-Ras4B^WT^ in MCF-10A and MCF-7 as well as K-Ras4B^G13D^ in MDA-MB-231 and MDA-MB-231RR by sequencing of exon 2 (**Supplementary Figure 2**).

First, we validated the oncogenic potential of the radioresistant cells as a suitable model. The results demonstrated that these cells exhibited a notably high migration and invasion capability (**Supplementary Figure 3**). Additionally, the radioresistant cells displayed a significantly higher migration velocity compared to their parental counterparts, indicating their heightened aggressiveness (**Supplementary Figure 3**). Likewise, MDA-MB-231 displayed a spindle-like, metastatic appearance with stress fibers, while MDA-MB-231RR differed by less stress fibers and exhibited numerous vacuoles or villi (**Supplementary Figure 4**). Thus, these findings establish MDA-MB-231RR cells as a model for radioresistant-TNBC with a heightened oncogenic potential and confirm their preservation of typical markers from their parental cell lineage.

**Figure 2:**
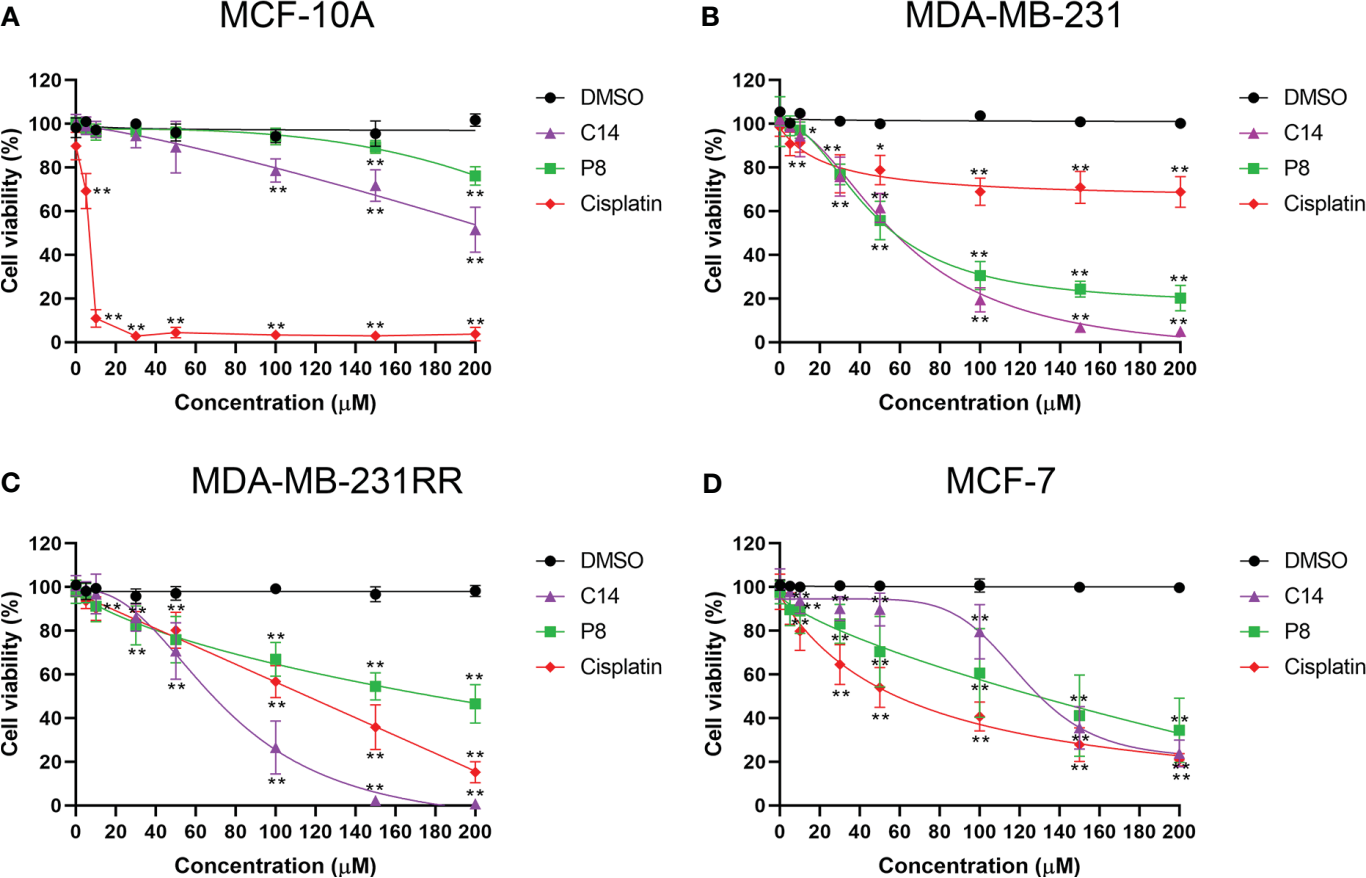
Compounds C14 and P8 decrease the cellular viability of breast cancer cells that express K-Ras4B^G13D^. **(A)** Dose–response curve of non-tumor MCF-10A cells treated with increasing concentrations of C14 and P8 compounds. **(B)** Dose–response curve of K-Ras4B^G13D^ in MDA-MB-231 cells treated with increasing concentrations of C14 and P8 compounds. **(C)** Dose–response curve of radioresistant MDA-MB-231RR cells treated with increasing concentrations of C14 and P8 compounds. **(D)** Dose–response curve of no mutant *KRAS* MCF-7 cells treated with increasing concentrations of C14 and P8 compounds. Concentration–response curves were evaluated after 48 h of exposure with increasing concentrations of C14 and P8 (0 to 200 µM) and show normalized percent activity for the individual doses. Cisplatin (0 to 200 µM) was used as a control, and maximal concentration of the vehicle (dimethyl sulfoxide (DMSO)) was evaluated. The line graph represents the mean means ± SEM from three independent experiments (*p< 0.05, **p< 0.01 compared to vehicle).

To determine whether C14 and P8 have a cytotoxic effect on breast cancer cell lines, an XTT assay was employed under experimental conditions for 24 h and 48 h. Antiproliferative effects of both compounds on MDA-MB-231 and MDA-MB-231RR cell lines were clearly observed. In contrast, a diminished cytotoxic effect was observed in the non-tumoral MCF-10A cell line (**Figure 2A–C**). Likewise, the IC50 values obtained for this cell line were greater than 200 µM at 24 h and 48 h post-treatment. According to statistical analyses using the “Sidak Bonferroni-type multiple comparisons” and “Multiple t-tests Holm–Sidak method”, concentrations higher than 100 µM and 150 µM are required to observe any effect of the compounds on cell viability in the control cells for C14 and P8, respectively. In comparison, in the TNBC and radioresistant TNBC cell lines, the compounds began to affect their viability at concentrations starting from 10 µM at 48 h. This suggests that C14 and P8 would not significantly reduce the viability of the non-tumoral cell line (**Figure 2A**).

In contrast, a significant cytotoxic effect of both compounds was observed on MDA-MB-231 (**Figure 2B**). Significance was noted from concentrations as low as 10 μM compared to the vehicle (DMSO) at 48 h. A similar pattern was observed in the radioresistant MDA-MB-231RR cell line, where the compounds exhibited a significant inhibitory effect on viability at 48 h, starting from 10 µM and 30 µM for P8 and C14, respectively (**Figure 2C**). Based on this information, C14 and P8 are able to promote diminution in cell viability of tumoral cells. The aforementioned statistical analyses suggested that the compounds demonstrate greater efficacy against triple-negative breast cancer cell lines with KRAS mutations compared to the non-tumorigenic control line. The improved effect of both compounds for the K-Ras4B^MUT^ form could be explained based on the more favorable ΔGbind values observed for the K-Ras4B^G13D^/PDE6δ complex. These data propose a preferential impact on aggressive breast cancer cells over non-tumoral cells.

To demonstrate the specific effects of C14 and P8 on cells expressing K-Ras4B^G13D^, their cytotoxic effect was assessed on the MCF-7 cell line, which expresses the wild-type form of K-Ras4B (27) (**Figure 2D**). The cytotoxic effect of C14 and P8 on MCF-10A, MDA-MB-231, MDA-MB-231RR, and MCF-7 cell lines was represented by the corresponding IC50 values (**Table 2**). As observed for MCF-10A cells, there was no significant effect on cell viability by both molecules (C14, IC50-48: 310.2 ± 210.0 μM; P8, IC50-48: 907.1 ± 291.0 μM) (**Table 2**). This result suggests that the compounds did not affect the growth of non-tumoral breast cells. However, in these control cells, the chemotherapeutic agent cisplatin reduced cellular viability by more than 90% at low concentrations (IC50-48: 5.9 ± 1.6 μM) (**Table 2**).

**Table 2:** IC50 values were calculated for several compounds in each cell line.

Compound	MCF-10A	MDA-MB-231	MDA-MB-231RR	MCF-7
IC50-24 (μM)				
C14	591.4 ± 210.0	91.1 ± 3.9	128.2 ± 2.6	174.2 ± 19.0
P8	1,146.8 ± 291.0	140.1 ± 33.5	163.3 ± 16.8	185.3 ± 15.3
Cisplatin	12.0 ± 0.0	352.1 ± 41.2	200.0 ± 0.0	150.3 ± 32.1
IC50-48 (μM)				
C14	310.2 ± 10.6	60.0 ± 6.6	70.6 ± 3.5	134.5 ± 10.5
P8	907.1 ± 219.2	63.2 ± 10.6	156.4 ± 28.4	103.6 ± 49.8
Cisplatin	5.9 ± 1.6	296.8 ± 34.8	103.6 ± 8.7	67.3 ± 20.6

IC50 values for 24 h (IC50-24) and 48 h (IC50-48) of each sample were calculated and shown in µM ± standard deviation of the mean.

In contrast, a cytotoxic effect of both compounds was observed in the triple-negative line MDA-MB-231. The IC50-48H values of 60.0 ± 6.6 µM and 63.2 ± 10.6 µM for C14 and P8, respectively, underscored their similar efficacy in suppressing TNBC cell viability (**Table 2**). This contrasted with cisplatin, for which an IC50-48 value nearly 40-fold higher was required when compared to MCF-10A cells (296.8 ± 34.8 μM) to achieve a comparable effect (**Table 2**). Likewise, the radioresistant MDA-MB-231RR cell line exhibited a comparable response to compound C14 with calculated IC50-48 values of 70.6 ± 3.5 µM (**Table 2**). In the case of P8, there was an effective response but with a higher IC50-48, which was 156.4 ± 28.4 µM (**Table 2**). Notably, the IC50-48 value for cisplatin, when evaluated in this context, was 103.6 ± 8.7 μM. Thus, these findings underscore the potential of C14 and P8 in both the parental and radioresistant TNBC cell lines. Their lower IC50-48 values compared to cisplatin suggest their promising role in addressing the challenges posed by drug resistance in TNBC treatment.

In comparison with cells that expressed the mutant form of KRAS gene, a major concentration of C14 was necessary to impact the cell viability of MCF-7 cells. The IC50-48 values obtained for this cell line were 134.5 ± 10.5 µM for C14 and 103.6 ± 49.8 µM for P8 (**Table 2**). It is important to note that despite this cell line being KRAS^wt^, a cytotoxic effect was observed, but the IC50-48 values were slightly higher than those obtained for mutant cell lines. Finally, MCF-7 cells exhibited more sensitivity to cisplatin with an IC50-48 value of 67.3 ± 20.6 µM (**Table 2**). Thus, our findings demonstrate that C14 and P8 have the potential to reduce the viability of breast cancer cell lines, with their primary efficacy against triple-negative and radioresistant cells and especially against the K-Ras4B mutant variants.

### 3.3 C14 and P8 compounds induce apoptosis in breast cancer cell lines

**Figure 3:**
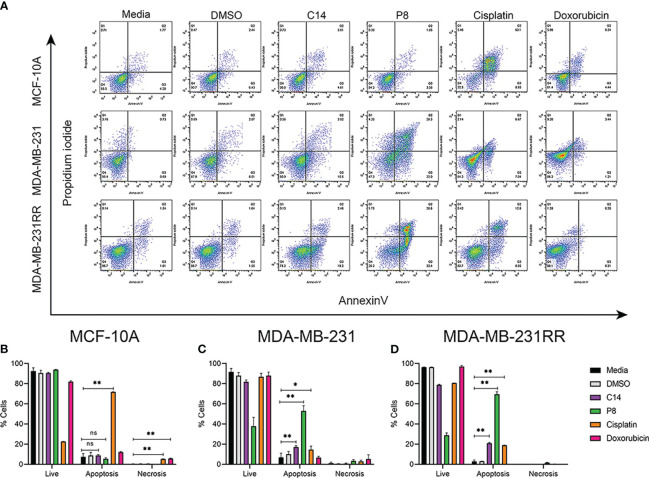
C14 and P8 compounds induce apoptosis in breast cancer cell lines. **(A)** Flow cytometric analysis diagram of compounds C14 and P8 for MCF-10A, MDA-MB-231, and MDA-MB-231RR cell lines. Evaluation of apoptosis/necrosis rate was conducted in a 24-h incubation period of compounds C14 and P8 at IC50-24 concentrations and Annexin V–propidium iodide staining. Cells kept in a growth medium or presence of vehicle (dimethyl sulfoxide (DMSO)) were included as control. Chemotherapeutic agents, 100 µM cisplatin and 0.5 µM doxorubicin for 24 h, were included as positive controls. **(B)** Graphical representation of percentage of live cells, apoptotic cells, and necrotic cells of MCF-10A in each condition. **(C)** Graphical representation of percentage of live cells, apoptotic cells, and necrotic cells of MDA-MB-231 in each condition. **(D)** Graphical representation of percentage of live cells, apoptotic cells, and necrotic cells of MDA-MB-231RR in each condition. Graphed results are means ± SEM from three independent experiments (*p< 0.05, **p< 0.01 compared to vehicle).; ns, not significant.

Mechanisms underlying these cytotoxic effects observed in TNBC and radioresistant cell lines were further investigated by flow cytometric assays by determination of percentage (%) of apoptosis cells (Annexin V) or % of necrosis (propidium iodide) following exposure to C14 and P8 being quantified employing the IC50-24 of each compound (**Figure 3A**). MDA-MB-231 cells showed that compound C14 promoted cell death *via* apoptosis in up to 18.52% of cells, with a minimal necrotic effect at 0.56%. Remarkably, P8 demonstrated even greater apoptotic potential, inducing cell death through apoptosis in 48.5% of cells and necrosis in 4.28% of the same cell line. In comparison, the chemotherapeutic agents cisplatin and doxorubicin promoted cell death *via* apoptosis in a lower percentage, 13.71% and 4.65% of cells, and greater necrosis in 2.14% and 9.20% of cells, respectively, compared to C14 and P8 (**Figure 3C**).

In the case of the MDA-MB-231RR cell line, C14 promoted cell death primarily through apoptosis in up to 21.62% of cells, with a minimal necrosis of only 0.13%. Compound P8 exhibited a remarkable capability to induce apoptosis in 72% of cells, while necrosis was observed in 1.75% of cells. Conversely, cisplatin induced apoptosis in up to 19.13% of cells, with necrosis in 0.40% of cells. Notably, the efficacy of doxorubicin was limited in radioresistant cells, inducing apoptosis in only 0.61% of cells and necrosis in 1.28% of cells (**Figure 3D**). According to these data, C14 and P8 reduced the growth of aggressive and radioresistant breast cancer cells, with a strong emphasis on apoptosis as the primary mode of action (57).

In contrast, neither C14 nor P8 induced significant cell death in normal breast MCF-10A cells, with viability at 91.1% and 94.3%, respectively. Conversely, cisplatin promoted apoptosis in over 70% and 5.46% of necrosis in normal breast cells. Doxorubicin also exhibited potent cytotoxic effects, leading to apoptotic cell death in up to 11.54% of cells and a necrotic cell death rate of 5.46% (**Figure 3B**).

These compelling findings not only reaffirm the cytotoxic potential of C14 and P8 but also shed light on their distinctive mechanisms of action. Particularly, P8 emerges as an inductor of apoptosis, an important pathway in the field of targeted cancer therapy. This revelation not only underscores the promise of C14 and P8 but also adds a significant dimension to their potential therapeutic applications, particularly in the challenging context of radioresistant cancer cells.

### 3.4 C14 and P8 compounds inhibit colony formation of breast cancer cell line

**Figure 4:**
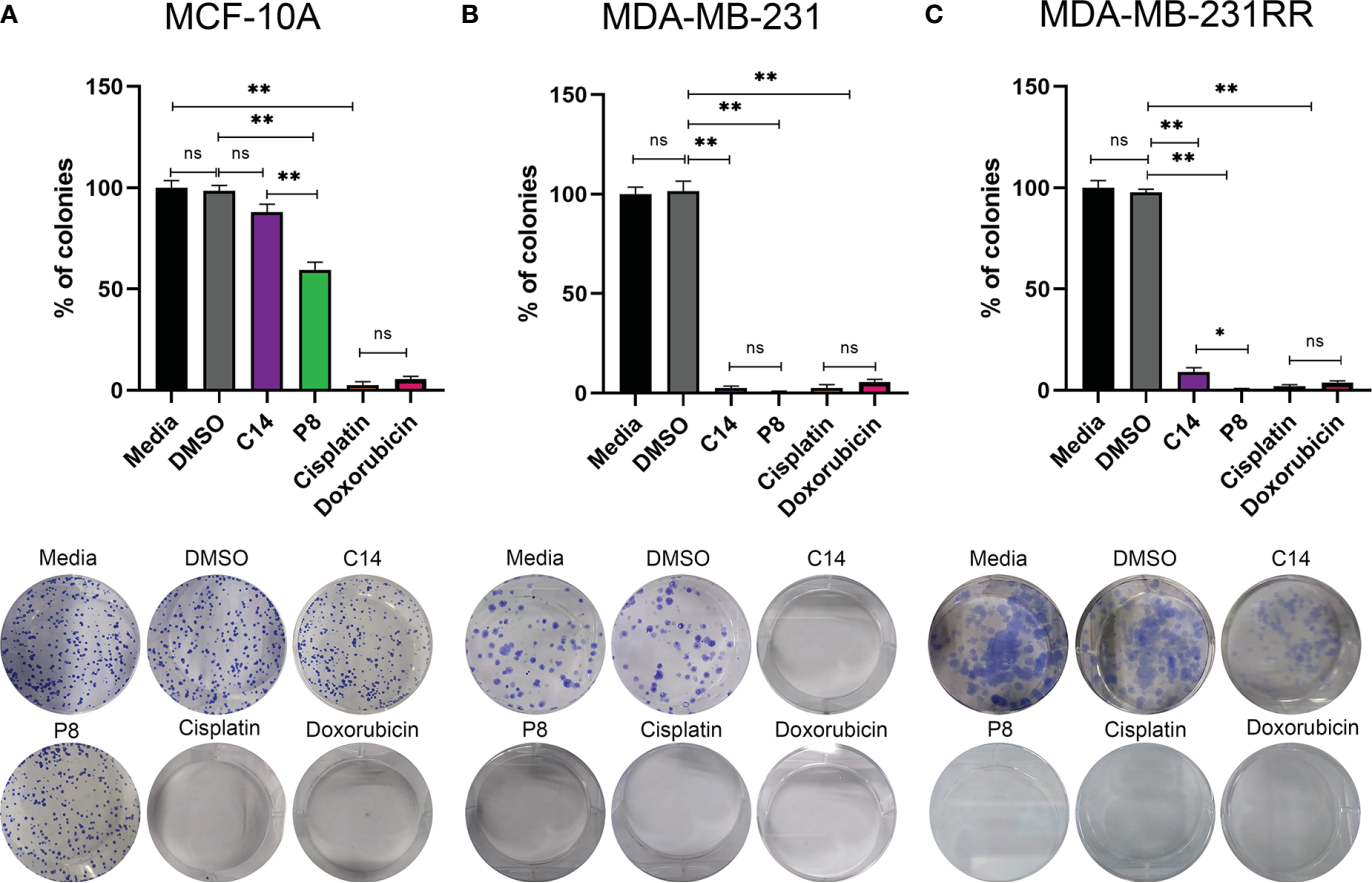
P8 inhibits the colony formation capability of triple-negative breast cancer (TNBC) and radioresistant cells. **(A)** Colony formation of MCF-10A cells after exposure to C14 and P8 compounds at IC50-48 value for 10 to 12 days. **(B)** Colony formation of MDA-MB-231 cells after exposure to C14 and P8 compounds at IC50-48 for 10 to 12 days. **(C)** Colony formation of MDA-MB-231RR cells after exposure to C14 and P8 compounds at IC50-48 for 10 to 12 days. Cells without treatment and cells treated with vehicle (dimethyl sulfoxide (DMSO) at 0.66%) were employed as controls. Cells treated with chemotherapeutic agents, 100 µM cisplatin and 0.5 µM doxorubicin, were employed as positive control. Bar charts show the percentage of counted colonies relative to control untreated cells and represent the means ± SEM of three independent experiments (*p< 0.05, **p< 0.01 versus control cells).; ns, not significant.

To assess the presence of cells capable of maintaining their proliferative capacity as colonies following exposure to the C14 and P8 compounds, clonogenic assays were conducted. After a period of 10 to 12 days, it was evident that in the case of MCF-10A, both compounds were not able to significantly impact the rate of growth of these non-tumoral cells. Specifically, C14 reduced the growth of 14% of colonies, and P8 reduced the growth of 48% of colonies (**Figure 4A**). Cisplatin significantly reduced the growth of 95% of colonies, while doxorubicin reduced the growth of up to 90% of colonies (**Figure 4B**).

In the case of MDA-MB-231 cells, both C14 and P8 led to an important decrease in colony formation, with reductions of up to 95% observed (**Figure 4B**). As expected, conventional chemotherapeutic agents, cisplatin and doxorubicin, were able to induce a significant reduction of colony growth (**Figure 4B**). Notably, P8 demonstrated a potent inhibitory effect on colony formation in MDA-MB-231-RR, with a reduction of up to 99%. In the case of C14, an inhibitory effect on colony formation with a reduction of up to 90% was observed in MDA-MB-231-RR cells (**Figure 4C**). Likewise, cisplatin (99% reduction in colony formation) and doxorubicin (90% reduction in colony formation) were both able to reduce colony growth (**Figure 4C**). In summary, the P8 compound efficiently inhibited colony formation in both MDA-MB-231 and the radioresistant cells, MDA-MB-231-RR. Although the C14 compound was effective against MDA-MB-231, nearly 10% of MDA-MB-231-RR colonies persisted.

These results highlight the significant implications of treatment-resistant cells, where C14 and P8 were effective in reducing colony formation in MDA-MB-231, particularly P8, which was the most effective in radioresistant MDA-MB-231-RR cells. In contrast, no significant impact was observed in MCF-10A with the C14 compound. However, P8 exhibited a 40% reduction in colony formation in this cell line. While this effect is less pronounced compared to its impact on cancer cell lines, it may be attributed to the intrinsic chemical characteristics of P8 within the complex K-Ras4B/PDE6δ that make it, in general, a more potent compound.

### 3.5 Significant reduction in Ras activation observed in the presence of compounds C14 and P8

**Figure 5:**
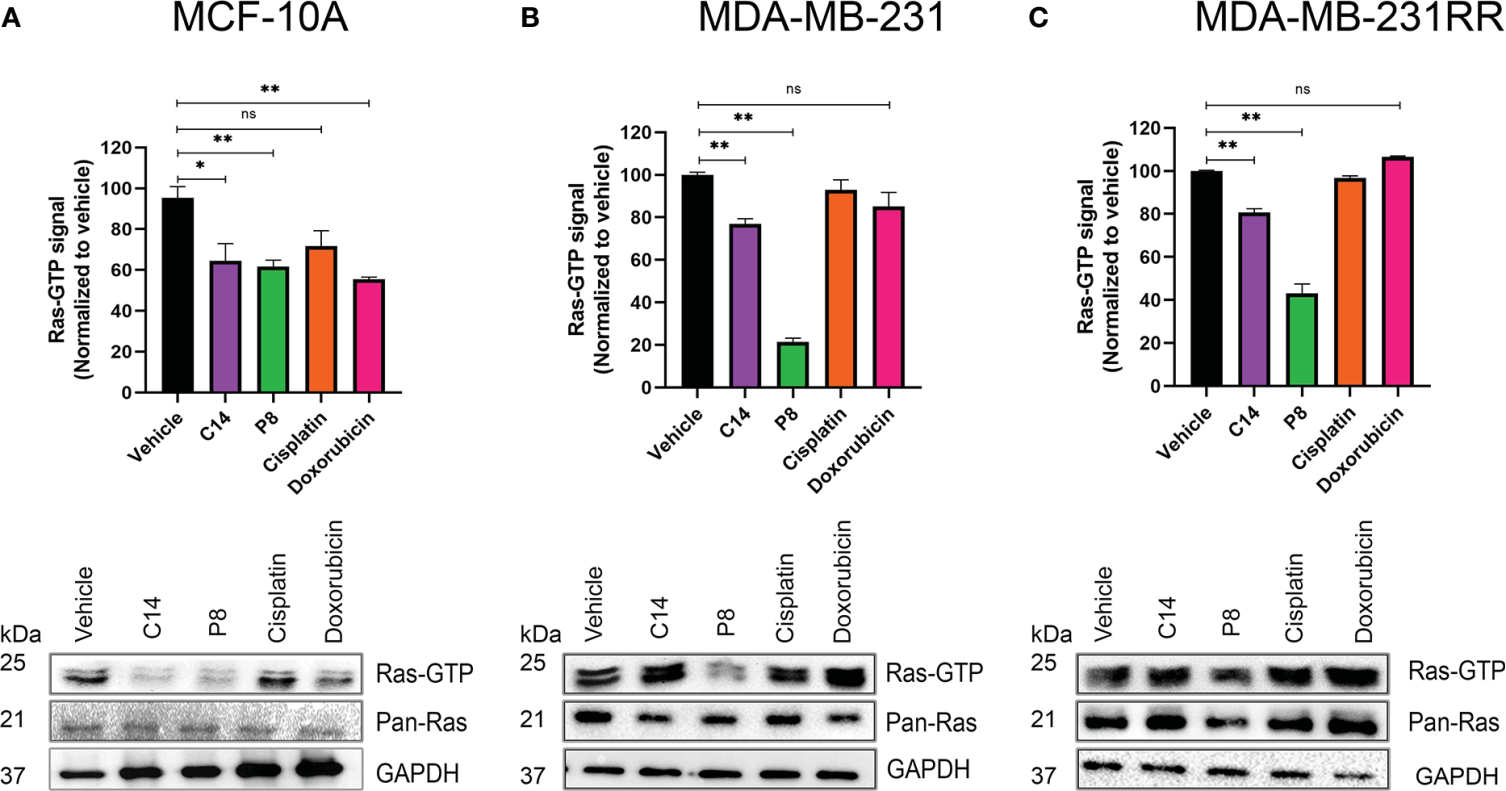
Amount of active Ras after cell exposure with C14 and P8. **(A)** MCF-10A cells that express the wild-type isoform of K-RAS4B. **(B)** MDA-MB-231 cells that express the mutant form K-Ras4B^G13D^ were treated with C14 and P8 for 3 h. **(C)** MDA-MB-231RR cells were also observed to have an important reduction of bound-GTP form of Ras, principally with P8 molecule. Dimethyl sulfoxide (DMSO) was employed as vehicle, and cisplatin and doxorubicin were employed as negative controls. Graphics represent the quantitative analysis of three independent assays. In blots, GAPDH was employed as loading control. Graphed results are means ± SEM from three independent experiments (*p< 0.05, **p< 0.01 compared to vehicle).; ns, not significant.

To further validate whether the specific impact of compounds C14 and P8 on TNBC and radioresistant cell lines is mediated through the reduction of Ras protein activation, pull-down assays were conducted within breast cancer cell lines, as illustrated in **Figure 5**.

In the MCF-10A cell line, exposure to C14 and P8 resulted in a reduction in Ras-GTP by approximately 40% (**Figure 5A**). In this case, also the compound doxorubicin reduced the amount of Ras-GTP by 40%, probably by a non-specific cell death effect. In contrast, cisplatin did not show an effect. Likewise, although the presence of C14 did not lead to a notable reduction in Ras-GTP levels (20%), compound P8 caused a dramatic reduction of over 80% in MDA-MB-231 cells (**Figure 5B**). These effects persisted in the radioresistant cells, with a decrease of approximately 20% in Ras-GTP levels with C14 and a more significant reduction of over 60% with P8 (**Figure 5C**).

According to the data shown, compounds C14 and P8 have an effect on the MCF-10A line at short exposure times (3 h). The reduction in K-Ras-GTP activity could be a result of the low activity levels of this molecule in non-tumor cells. On the contrary, compound C14 did not show a substantial effect on K-Ras activity in MDA-MB-231 and MDA-MB-231RR. This apparent lower effectiveness could be explained by two aspects: the abundant activity levels of this GTPase in cancer cell lines and the short exposure time to the compounds. Probably, C14 requires longer periods of time to affect its target. In the case of P8, a potent reduction in Ras-GTP levels is observed in both tumoral cell lines. This could be because this molecule exhibits a more powerful effect in a short time due to its chemical characteristics.

### 3.6 Compounds C14 and P8 decrease Ras activity and inhibit AKT and ERK phosphorylation in MDA-MB-231 and MDA-MB-231RR cells

**Figure 6:**
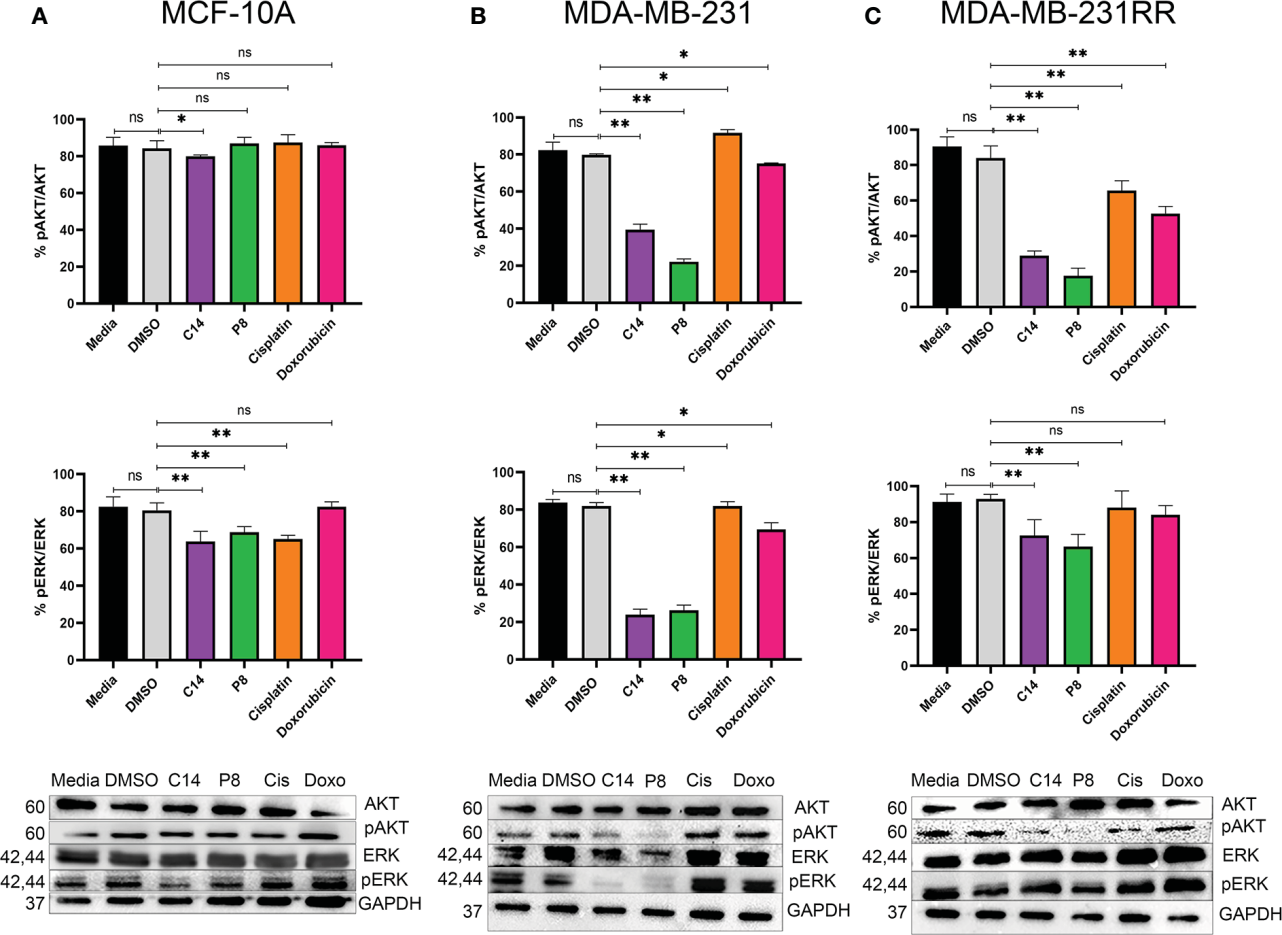
Compounds C14 and P8, decrease AKT and ERK phosphorylation. **(A)** Representative immunoblots of whole protein extracts from MCF-10A, plotted against total AKT and ERK proteins and phosphorylated AKT and ERK forms. **(B)** Representative immunoblots of whole protein extracts from MDA-MB-231, plotted against total AKT and ERK proteins and phosphorylated AKT and ERK forms. **(C)** Representative immunoblots of whole protein extracts from MDA-MB-231RR, plotted against total AKT and ERK proteins and phosphorylated AKT and ERK forms. Cells were treated with C14 and P8 at IC50-48, Cisplatin at 100 µM and Doxorubicin at 0.5 µM for 3 h. Cells kept in growth media or media plus vehicle (DMSO), were employed as control. After pre-treatment, cells were stimulated with 100 ng/ml EGF for 10 minutes to promote AKT and ERK activation. GAPDH was plotted with specific antibodies as control. Graphed results are means ± SEM from three independent experiments of pAKT and pERK (*p < 0.05, **p < 0.01 compared to vehicle).; ns, not significant.

Furthermore, it has been widely reported that molecules downstream of K-Ras4B, such as pAKT and pERK, are related to the signaling pathways involved in cell survival and differentiation. In order to ascertain whether the effect of the C14 and P8 compounds on the Ras activation negatively impacts the activation of critical downstream molecules regulated by K-Ras4B^G13D^, the levels of pAKT and pERK activation in MCF-10A, MDA-MB-231, and MDA-MB-231RR cells were determined by Western blotting (**Figure 6**). Densitometric analysis of the blots revealed in the graph that in MCF-10A, there was a reduction in the phosphorylation levels of pERK (40%) and pAKT (25%) after exposure to C14 and P8 (**Figure 6A**, upper and middle panel). In MDA-MB-231, C14 and P8 exhibited a significant impact by diminishing pERK up to 70% with both compounds (**Figure 6B**, middle panel). Likewise, there was a substantial reduction in the levels of pAKT in these cells treated with P8 (up to 70%) and C14 (up to 60%) (**Figure 6B**, upper panel). In MDA-MB-231-RR cells, C14 induced a reduction of approximately 30% in pERK, while a 35% reduction in pERK was observed with P8 (**Figure 6C**, middle panel). However, there was a marked reduction in pAKT levels with both compounds (>70%) (**Figure 6C**, upper panel).

These findings demonstrate that C14 and P8 reduce the activity of signaling pathways regulated by K-Ras, primarily *via* pAKT, in tumoral and radioresistant breast cancer cell lines. In the case of MDA-MB-231, there was a clear reduction in pERK after exposure to C14 and P8. Conversely, a non-clear effect was observed in MDA-MB-231RR, likely due to its more aggressive genotype. However, the clear impact of both compounds on pAKT suggests their potential use as therapeutic options against TNBC and radioresistant TNBC.

### 3.7 Radioresistant-related signaling pathway effect and induction of cell cycle arrest in human radioresistant breast cancer cells by compounds

To determine the effects observed of compounds C14 and P8 on the AKT activation on radioresistant cells, the pathway associated with cell proliferation *via* K-Ras4B negatively impacts the radiation resistance acquisition pathways regulated by this kinase, particularly the AKT/GSK3β/cyclin D1 pathway. The protein levels of cyclin D1 and Glycogen Synthase Kinase-3β (GSK3β) were evaluated in MCF-10A, MDA-MB-231, and radioresistant MDA-MB-231RR cell lines.

**Figure 7:**
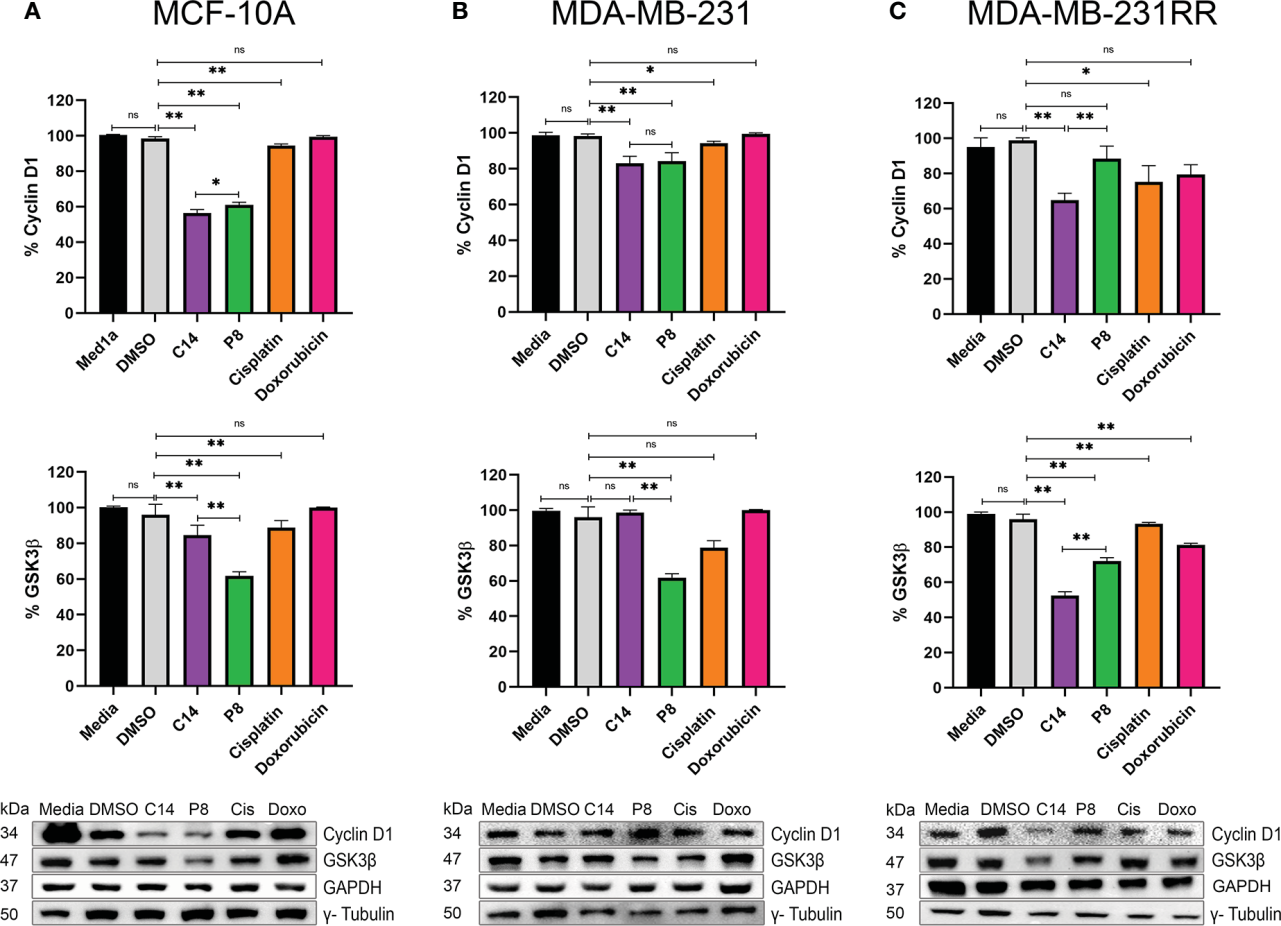
Compounds C14 and P8, decrease GSK3β and Cyclin D1. **(A)** Representative immunoblots of whole protein extracts from MCF-10A, plotted against total Cyclin D1 and GSK3β proteins. **(B)** Representative immunoblots of whole protein extracts from MDA-MB-231, plotted against total Cyclin D1 and GSK3β proteins. **(C)** Representative immunoblots of whole protein extracts from MDA-MB-231RR, plotted against total Cyclin D1 and GSK3β proteins. Cells were treated with C14 and P8 at IC50-24, Cisplatin at 100 µM and Doxorubicin at 0.5 µM for 3 h. GAPDH and γ- Tubulin was plotted with specific antibodies as loading control. Quantitative representation of 3 independent immunoblots studies, are shown in the upper and middle graphs, respectively. Graphed results are means ± SEM from three independent experiments of pAKT and pERK (*p < 0.05, **p < 0.01 compared to vehicle).; ns, not significant.

This method was adopted, considering that human tumor cells develop radioresistance when exposed to fractionated X-ray radiation (FR) (34, 58). Likewise, during this process, cyclin D1 is overexpressed to potentially enhance tumor cell proliferation (59). Densitometric analysis of blots showed that in MCF-10A, there was a 40% reduction in cyclin D1 protein after exposure to C14 and P8, compared to controls (media and DMSO) and chemotherapeutic agents (cisplatin and doxorubicin) (**Figure 7A**, upper panel). In MDA-MB-231, both molecules reduced the expression of cyclin D1 by 20%. There was no significant effect in controls and doxorubicin. On the contrary, cisplatin reduced the expression of cyclin D1 by 10% (**Figure 7B**, upper panel). In radioresistant MDA-MB-231-RR cells, there was a significant reduction in cyclin D1 with the C14 compounds (40%). However, P8 diminished the expression of cyclin D1 by 18%. Finally, cisplatin and doxorubicin reduced the presence of cyclin D1 by 20% (**Figure 7C**, upper panel). According to the results, cyclin D1 levels in tumoral cells were consistently higher than those observed in control cells, and both compounds showed a potent effect. This difference could be attributed to the elevated levels of cyclin D1 in tumor cell lines (60).

In contrast, although the impact of ionizing radiation on GSK3β is multifaceted, it could be associated with the expression of cyclin D1 (61). In this case, administration of C14 in MCF-10A reduced the amount of GSK3β by 10%. By P8, the reduction was 40% (**Figure 7A**, middle panel). By MDA-MB-231, C14 did not show an effect on this molecule. In contrast, in this cell line, the P8 compound reduced the expression of GSK3β principally by 40% (**Figure 7B**, middle panel). Finally, in MDA-MB-231RR, P8 and C14 respectively reduced 25% and 50% the expression of GSK3β (**Figure 7C**, middle panel). These data suggest that in MDA-MB-231RR cells, C14 principally inhibits the effect of GSK3β/cyclin D1 molecules probably *via* AKT. However, these aspects must be studied in greater depth.

**Figure 8:**
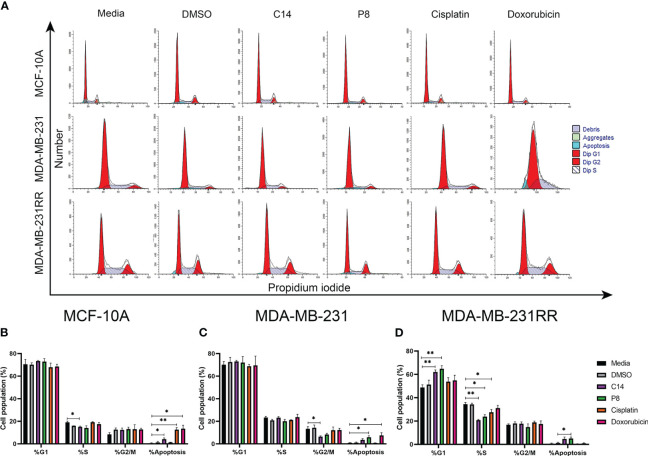
Compounds induced G0/G1 cell cycle arrest and apoptosis in radioresistant cells. **(A)** A representative histogram of propidium iodide (PI) staining in breast cancer (BC) cells. **(B)** MCF-10A cell population proportion in each cell cycle phase. **(C)** MDA-MB-23 cell population proportion in each cell cycle phase. **(D)** MDA-MB-231RR cell population proportion in each cell cycle phase. After 3 h of treatment with C14, P8, cisplatin, and doxorubicin. Graphed results are means ± SEM from three independent experiments of pAKT and pERK (*p< 0.05, **p< 0.01 compared to media).

To investigate the mechanism behind the anticancer activity of C14 and P8 and their increased sensitivity in radioresistant breast cancer cells, we analyzed the cell cycle distribution using flow cytometry. As shown in **Figure 8**, the majority of MCF-10A control cells remained in the G1 phase even after treatments (**Figures 8A, B**). Treatment with doxorubicin and cisplatin showed a similar number of cells arrested in the S phase (**Figures 8A, B**). Additionally, we observed that most of the MDA-MB-231 cells were arrested in the G0/G1 phase compared to the control (**Figures 8A, C**). The percentage of cells in G2/M decreased from 10.0% to 6.4% and 8.2% after treatment with C14 and P8, respectively, but increased after treatment with doxorubicin (**Figures 8B, D**). MDA-MB-231RR cells were also arrested in the G0/G1 phase compared to the control after treatment and exhibited a decrease in the S phase from 35.6% to 20.7% and 26.4% after treatment with C14 and P8, respectively (**Figures 8C, D**). Notably, there were no significant changes in the G2/M phase of the MDA-MB-231RR cells following treatment. Additionally, a slight increase in apoptotic cells was observed in the RT group compared to the control.

The arrest of TNBC and radioresistant TNBC cells in the G0/G1 phase and the reduction of cells in the S phase following treatment with the C14 and P8 compounds, principally in the context of MDA-MB-231RR, imply a disruption in the cell cycle progression. It is a mechanism often targeted in cancer therapy to inhibit the uncontrolled growth of cancer cells particularly concerning treatment-resistant cells.

### 3.8 Inhibition of tumor growth in a radioresistant breast cancer xenograft mouse model by compounds C14 and P8

**Figure 9:**
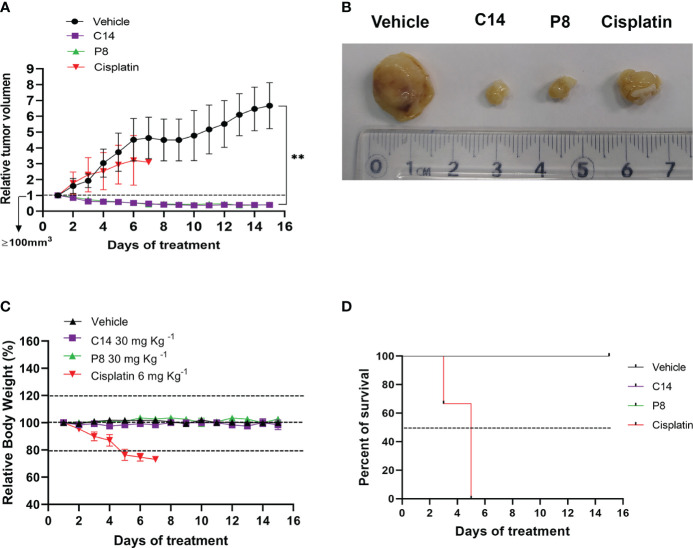
Inhibition of tumor growth by compounds C14 and P8 in a xenograft model of radioresistant MDA-MB-231RR cells. **(A)** The relative tumor volume was evaluated over a 15-day treatment period. **(B)** Representative images depicting tumor sizes. **(C)** Body weight measurements of mice were recorded during the treatment period. Nu/Nu mice were treated with the following: vehicle (10% dimethyl sulfoxide (DMSO) and 0.05% carboxy methyl cellulose), C14 and P8 at 30 mg/kg, or cisplatin at 6 mg/kg, administered daily by intraperitoneal injection (n = 5 for DMSO, n = 5 for C14, n = 5 for P8, and n = 5 for cisplatin). Changes in tumor volume are given in relation to the initial volume before treatment (the dotted line indicates the initial size of the tumor ~100 mm^3^). **(D)** Survival curve throughout the treatment period. Mice treated with cisplatin at 6 mg/kg did not survive beyond 6 days. The line graph represents the mean and SD (**p< 0.01).

Based on the effectiveness of C14 and P8 obtained *in vitro*, specifically the reduction in the proliferation and growth of radioresistant cells, the antitumor activity of both molecules was evaluated using an *in vivo* model. To accomplish this, highly aggressive radioresistant cancer cells, MDA-MB-231 RR, were subcutaneously inoculated into female Nu/Nu mice to closely monitor tumor growth. The different treatments were administered *via* intraperitoneal (i.p.) injection daily for 2 weeks (**Figure 9A**). The results showed a 40.0% reduction in tumor size in mice treated with compound C14 and a 41.6% reduction in those treated with P8. It is crucial to note that these treatments, administered at 30 mg/kg, were well tolerated by the mice, with no weight loss or impact on their overall survival rates observed. Conversely, mice treated with cisplatin (6 mg/kg) experienced over a 20% weight decrease within the first 5 days of treatment, necessitating ethical considerations for the wellbeing of the animals in this group (**Figures 9C, D**). All data emphasized not only the non-toxic nature of compounds C14 and P8 but also their specific anti-neoplastic effects, especially against breast cancer cells inhibiting tumor growth (**Figures 9A, B**). Likewise, it highlights the specific effect of these compounds over radioresistant cells.

### 3.9 Inhibition of proliferation and expression of CD31 by compounds C14 and P8 in a radioresistant TNBC mouse model

**Figure 10:**
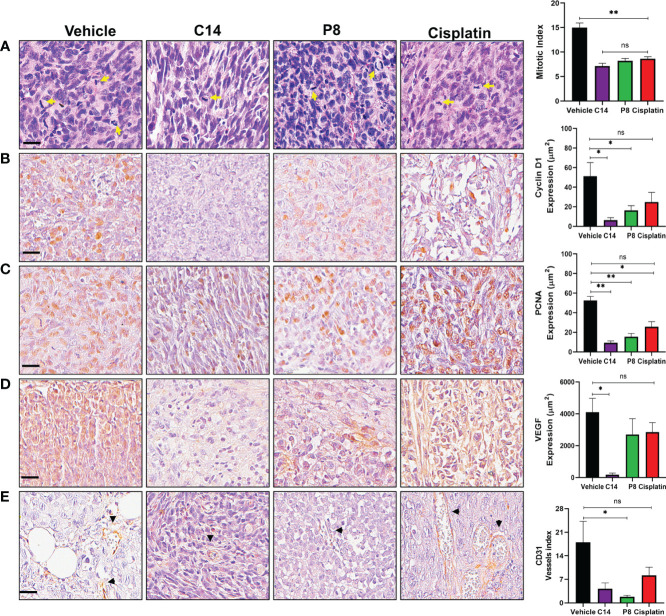
Inhibition of proliferation and angiogenesis by compounds C14 and P8 in a radioresistant triple-negative breast cancer (TNBC) mouse model. **(A)** Representative images of breast cancer tumors with H&E staining and evaluation of the mitotic index. Yellow arrows indicate cells undergoing mitosis. **(B)** Immunostaining and analysis of cyclin D1. **(C)** Immunostaining and analysis of PCNA nuclear expression. **(D)** Analysis of VEGF expression assessed *via* immunohistochemistry. **(E)** Quantification of blood vessels using CD31 immunostaining. Black arrowheads represent blood vessels. Scale bar, 20 µM. The data are presented as the mean ± SEM, n = 5 per group (*p< 0.05, **p< 0.01).; ns, not significant.

According to the data presented above, C14 and P8 have shown effects on cell cycle progression by inducing arrest in the G0/G1 phase. To comprehensively evaluate and support these observations, various markers of proliferation and angiogenesis were assessed using an *in vivo* model through microphotography of IHC of tumor samples (**Figure 10**). To enhance the quality of the analysis of the images, a digital pathology system was employed. With this system, it was possible to evaluate the expression of proliferation and angiogenesis markers with transparency and consistency. The digital pathological analysis of immunostainings demonstrated the reduction in proliferation markers, indicated by a lower mitotic index in the H&E staining in both C14 and P8 treatment groups when compared to the control group (**Figure 10A**). Likewise, C14 promotes the inhibition of cyclin D1 (8 μm^2^), PCNA (10 μm^2^), and VEGF (100 μm^2^), highlighting its potential in reducing key markers associated with DNA synthesis during replication and the control of cell cycle progression for proliferation (62). In contrast, P8 and cisplatin also reduced the expression of cyclin D1 (20 μm^2^ and 25 μm^2^, respectively), PCNA (18 μm^2^ and 22 μm^2^, respectively), and VEGF (3,000 μm^2^ in both cases) (**Figures 10B–D**). These data support the inhibition of cell cycle progression by C14 and P8 observed in cell lines and mouse models.

Likewise, the expression of CD31, an endothelial protein related to the restoration and maintenance of blood vessels and angiogenesis, was evaluated (63). In this case, both compounds but principally P8 decreased the expression of CD31 (**Figure 10E**). This finding holds significance, as CD31 plays a crucial role in the formation of fresh blood vessels and functions as an indicator of angiogenesis (64). Taken together, the outcomes indicate that compounds C14 and P8 could reduce the expression of molecules related to the formation of new blood vessels in radioresistant TNBC. However, the specific role of both compounds during angiogenesis should be addressed more thoroughly.

## 4 Discussion

BC is a highly prevalent and deadly disease among women worldwide. Its aggressive nature is characterized by several clinical manifestations, high cellular diversity within tumors, and distinct gene expression patterns. As a result, numerous treatment approaches have been developed to reduce the negative effects of this complex disease. However, TNBC is of particular concern due to limited treatment options, typically limited to chemotherapy or radiotherapy, as it lacks the hormone receptors and targeted therapies effective in other breast cancer subtypes (65). This aggressive nature and resistance to therapies highlight the urgent need for ongoing research and the development of novel treatment strategies to improve outcomes for TNBC or radioresistant TNBC patients and reduce mortality.

To address this need, the focus of the present study is to evaluate the cytotoxic effects of compounds C14 and P8. As mentioned above, to the best of our group’s knowledge, there is only one study reporting the specific types of K-Ras4B mutations in patients with BC (24). Also, the unique mutant form of K-Ras4B reported in cellular models of BC is G13D (27). Likewise, few reports have shown a low frequency of mutant forms of K-Ras4B in BC (7%–12%) (29). Based on this information, it is clear that there is a need to carry out a more in-depth evaluation to determine the real type and frequency of K-Ras4B mutations in BC.

To achieve our goal, the cell lines MDA-MB-231 and MDA-MB-231RR were employed. These cell lines are particularly relevant because they exhibit TNBC characteristics and express the K-Ras4B^G13D^ mutation. In this case, clear effectiveness was observed by C14 and P8 as antitumoral agents in TNBC cells that express the mutant form of G13D. This is achieved through the stabilization of the molecular complex of K-Ras4B^G13D^/PDE6δ and the reduction of associated signaling pathways (31–33, 66).

With this information, we propose that C14 and P8 could be considered as potential therapeutic options for TNBC or for TNBC stages that develop resistance to conventional therapies, especially in cases with a poor prognosis and limited therapeutic alternatives. Additionally, we propose that the effectiveness of both compounds against TNBC is not limited to cells that express the G13D mutation. This observation is supported by previous reports in which the antitumoral effects of C14 and P8 were determined over different mutations of K-RAS4B, such as G12D, G12C, and G12V (33). The importance of this multiple effect is clear considering the lack of information about the frequency and type of mutation of K-Ras present in TNBC.

Selectivity of C14 and P8 over the mutant forms of K-Ras4B^G13D^ was further demonstrated through *in silico* analysis. These results, as indicated by the binding free energy (ΔGbind), show that both the C14 and P8 compounds enhance the affinity of the mutated K-Ras4B^G13D^ variant for PDE6δ compared to the K-Ras4B^wt^/PDE6δ counterpart. Furthermore, our predictive modeling indicated a greater efficiency of P8 in stabilizing the K-Ras4B^G13D^/PDE6δ complex when compared to compound C14. This increased ability to stabilize the complex might potentially result in blocking the abnormal activation of the K-Ras4B^G13D^ signaling pathway, subsequently inducing apoptosis in breast cancer cells (67). These findings align with previous studies, demonstrating that this compound family possesses the ability to stabilize the K-Ras4B^mut^/PDE6δ complex, irrespective of the aggressiveness state, or the development of resistance against conventional therapies. This underscores their potential for therapeutic application in advanced stages of breast cancer.

Given the distinct nature of K-Ras4B^G13D^, characterized by heightened affinity and GDP-to-GTP exchange compared to K-Ras4B^G12D^, where intrinsic GTPase activity remains inhibited, effectively entrapping K-Ras4B in a constitutively active state, we postulate two potential mechanisms underlying the influence of compounds on the K-Ras4B^G13D^/PDE6δ complex. These mechanisms involve the hindrance of complex dissociation and K-Ras4B anchoring to the plasma membrane. Alternatively, due to the compounds’ demonstrated affinity for both K-Ras4B protein and GDP, they might perturb the activity of guanine exchange factor (GEF) proteins, thereby impeding the GDP-to-GTP exchange process (68). In either scenario, the outcome is the inhibition of protein activation and KRAS-dependent signaling pathways through the binding of the K-Ras4B^MUT^/PDE6δ complex with C14 or P8.

Conversely, it is noteworthy that both compounds exhibit interaction with nearly identical amino acid residues. This observation implies that concurrent administration of C14 and P8 to the complex could lead to the establishment of competitive interactions among the analyzed components. The results of the MD assay predict that the separate use of compounds C14 and P8 should independently stabilize the K-Ras4B^G13D^/PDE6δ complex, consequently exerting a detrimental impact on the activation of K-Ras signaling pathways within mutant breast cancer cell lines.

The specificity of both compounds for the mutated K-Ras4B^G13D^/PDE6δ complex, in comparison to the K-Ras4B^WT^/PDE6δ counterpart, was further demonstrated by cytotoxic assays. In them, significantly higher IC50 values were observed in the non-tumoral MCF-10A cell line compared to TNBC cell lines and the radioresistant TNBC cell line, which showed reduced IC50 values.

In MDA-MB-231RR cells particularly, both molecules C14 and P8 displayed potent cytotoxic effects, indicating their potential to reduce the viability of radioresistant TNBC cell lines. This is particularly significant, considering the highly aggressive phenotype associated with the acquisition of radioresistance (69). Moreover, the unique impact of C14 and P8 on mutant TNBC cell lines compared to non-tumoral cells underscores their potential as selective anticancer treatments, minimizing side effects. In contrast, conventional chemotherapy agents displayed substantial cytotoxicity in non-tumoral cells, emphasizing the potential advantages of C14 and P8 over traditional treatments (70).

This study also validates the distinct influence of these compounds on KRas4B protein function, as evidenced by the reduction in Ras-GTP levels and GTPase effectors observed in breast cancer cells when exposed to C14 and P8. The binding to K-Ras^G13D^/PDE6δ reduces the activation of Ras proteins, making these compounds good candidates for targeted therapies against breast cancer, particularly in TNBC or in patients that present resistances to conventional therapies, in which dysregulation in the Ras pathway generally occurs and plays a central role in tumorigenesis (14). Another crucial aspect to consider is the mechanism by which these compounds trigger cell death. While both C14 and P8 initiate apoptosis in the TNBC cell line and radioresistant TNBC cells, P8 demonstrates superior effectiveness as mentioned in earlier reports (33). It is also notable that these molecules show reduced necrosis and low cytotoxicity in non-tumoral cells. In contrast, conventional chemotherapy agents led to substantial cell death, including both apoptosis and necrosis, emphasizing the potential of C14 and P8 as therapies for TNBC and radioresistant cells, focusing on apoptosis-driven cell death mechanisms with minimized inflammatory effects.

The apoptosis-driven cell death mechanism elicited by C14 and P8 is associated with their negative impact on Ras activity, which directly impacted K-Ras-dependent pathways, including AKT and ERK (71). These experimental findings suggest that both compounds could be associated with survival, cell cycle progression, and cell growth in MDA-MB-231 and MDA-MB-231RR cells. Likewise, neither molecule causes reduced signaling pathway inhibition in the MCF-10A cell line. Interestingly enough, MDA-MB-231 cells presented more dependency and effectiveness at the ERK pathway, while MDA-MB-231RR presented higher dependency on the AKT pathway. Although these observations highlight the complex interplay between these compounds and the signaling pathways within different cellular contexts, it is important to mention that both molecules do not lose their effectiveness as antitumor agents in advanced stages of breast cancer.

The administration of C14 and P8 resulted in the observed loss of clonogenic capability in MDA-MB-231 cells. Nonetheless, MDA-MB-231RR cells presented a low grade of resistance against C14 but not P8. This observation is important considering that MDA-MB-231RR is a radioresistant cell line with a more aggressive phenotype, such as high proliferation rates and high migration, velocity, and invasion phenotypes. According to previous reports, exposure to ionizing radiation evokes a higher proliferation rate and chemoresistance that can also be attributed to the presence of a small population of cancer stem cells (CSCs) (69, 72). In this case, despite not quantifying the number of CSCs in MDA-MB-231RR, the small number of cells capable of surviving the administration of C14 could be attributed to this kind of cell based on the more aggressive phenotype of MDA-MB-231RR. In contrast, it is important to mention that in the case of compound P8, a total inhibition of the clonogenic capacity of the MDA-MB-231RR cell line was observed. These data position this compound as a possible molecule capable of inhibiting the effects of CSC. However, it is important to highlight that the C14 and P8 compounds were completely effective in inhibiting the clonogenic ability of MDA-MB-231 cells. This effect could be attributed to the presence of fewer CSCs in this cell line. In conclusion, both molecules are highly effective against TNBC. However, P8 demonstrates greater potency in the context of more aggressive behavior, advanced stages, and resistance states, which could be attributed to its enhanced capability to eradicate CSC. This is associated with its high affinity to inhibit the activity of the K-Ras4B^G13D^/PDE6δ complex.

According to the information presented in **Figures 7C** and **10B**, C14 reduces the amount of GSK3β/cyclin D1. In this case, it is proposed that this effect is attributable to the inhibition in the activity of the K-Ras4B^G13D^/PDE6δ complex and its molecular effector AKT. The reduction in this signaling pathway has a direct impact on the acquisition of a radioresistant phenotype. In this context, reports have shown that a fraction dose of ionizing radiation leads to radioresistance. A similar protocol was applied for established long-term FR cells MDA-MB-231RR (34). Thus, the acquired radioresistant phenotype is long-lasting and possibly irreversible as a result of the constitutive activation of AKT/GSK3β/cyclin D1/Cdk4 pathway, which is induced by a positive feedback loop mediated through the cyclin D1 overexpression, which triggers the development of radioresistance in tumor cells (58). Considering this information, it is suggested that C14 could reduce the activity of the AKT/GSK3β/cyclin D1 axis and potentially prevent the acquisition of radioresistance when it is administered before radiotherapy. This hypothesis requires more comprehensive evaluations.

Additionally, cell cycle analysis revealed the impact of the C14 and P8 compounds on the G0/G1 phase. The C14 compound was effective in inhibiting the progression of the S phase, resulting in the inhibition of cell growth and the induction of apoptosis in radioresistant breast cancer cells. Conversely, treatment with P8 resulted in a substantial increase in the number of cells in the G0/G1 phase and a concurrent decrease in the number of cells in the S phase in MDA-MB-231RR cells. These observations highlight the distinctive roles of C14 and P8 in influencing the cell cycle dynamics of breast cancer cells, particularly in the context of radioresistant cells.

Taking into consideration the limited therapeutic alternatives available for TNBC patients who have developed radioresistance, and in addition to the data that have demonstrated compounds C14 and P8 as antitumor molecules, their efficiency was evaluated in an *in vivo* model. The model employed involved female Nu/Nu mice and radioresistant MDA-MB-231RR cells to induce tumors. The treatments with C14 and P8 showed a significant reduction in tumor size, with a 40.0% reduction in C14-treated mice and a 41.6% reduction in P8-treated mice. Importantly, these treatments were well-tolerated and non-toxic, in contrast to cisplatin, which induced significant weight loss. These results underscore the non-toxic nature of C14 and P8 and emphasize their specific antineoplastic properties against breast cancer cells, especially radioresistant cells.

To verify the effects of the C14 and P8 compounds on cell progression in *in vitro* models, the mitotic index was determined in *in vivo* models through H&E staining. This analysis showed that the administration of C14 and P8 resulted in a significant reduction in cell proliferation of radioresistant TNBC cells, supporting the findings observed in *in vitro* models. These results were confirmed by observing a significant reduction in the expression of cyclin D1 and PCNA, which are essential regulators of the cell cycle. Likewise, C14 and P8 were found to influence angiogenesis-related biomarkers, specifically VEGF and CD31, in radioresistant TNBC tumors. A decrease in VEGF levels was observed in the C14-treated group compared to the control. Additionally, CD31, a marker of neo-vascularization, exhibited a reduction in the P8 group. This observation opens the door to exploring the effect of both compounds during the angiogenesis process.

Finally, while the cytotoxic effects of compounds C14 and P8 in TNBC and radioresistant TNBC cells were demonstrated, there are several considerations that must be taken into account for their potential use in the clinical setting. This includes the need for extensive clinical trials to validate the efficacy and safety of these compounds in humans. Long-term studies are essential, focusing on potential resistance development, recurrent tumor growth, and the sustainability of the compounds’ efficacy over extended treatment periods. Further investigation into the detailed molecular mechanisms underlying the inhibitory effects of these compounds over K-Ras4B^G13D^ is also required.

In conclusion, the present work shows a comprehensive view of the molecular changes induced by C14 and P8 in TNBC and radioresistant TNBC cells. Both molecules are effective in stabilizing and inhibiting the action of the mutant form of K-Ras4B, K-Ras4B^G13D^, and its association with its membrane transporter, PDE6δ. The antineoplastic evaluation of these compounds demonstrates that both molecules preferentially affected K-Ras4B mutated forms. Furthermore, C14 and P8 influence critical signaling pathways related principally to cell survival and cell cycle regulation to reduce cell proliferation.

## Data availability statement

The authors acknowledge that the data presented in this study must be deposited and made publicly available in an acceptable repository, prior to publication. Frontiers cannot accept an article that does not adhere to our open data policies.

## Ethics statement

The animal study was approved by Ethics approval and consent to participate The Center for Research and Advanced Studies (CINVESTAV) fulfill the standards of the Mexican Official Norm (NOM-062-ZOO-1999) “Technical Specifications for the Care and Use of Laboratory Animals” based on the Guide for the Care and Use of Laboratory Animals “The Guide”, 2011, NRC, USA with the Federal Register Number # BOO.02.08.01.01.0095/2014, awarded by the National Health Service, Food Safety and Quality (SENASICA) belong to the Animal Health Office of the Minister of Agriculture, Livestock, Rural Development, Fisheries and Food (SAGARPA), an organization that verifies the state compliance of such NOM in Mexico. The Institutional Animal Care and Use Committee (IACUC) from the CINVESTAV as the regulatory office for the approval of research protocols involving the use of laboratory animals and in fulfillment of the Mexican Official Norm resolved: TO APPROVE THE FOLLOWING RESEARCH PROJECT TITLED: “EVALUATION OF COMPOUNDS C14 AND P8 IN PRECLINICAL STAGES ON MAMMOSPHERES OF RADIORESISTANT AND NON-RADIORESISTANT CELLS”. ID Animal use protocol number: 0319-21. The study was conducted in accordance with the local legislation and institutional requirements.

## Author contributions

DC: Conceptualization, Formal analysis, Investigation, Methodology, Writing – original draft, Writing – review & editing. AA: Formal analysis, Investigation, Methodology, Resources, Writing – review & editing. SH: Formal analysis, Methodology, Writing – review & editing. MM: Formal analysis, Methodology, Writing – review & editing. MB: Formal analysis, Methodology, Resources, Writing – review & editing. AR: Formal analysis, Investigation, Resources, Software, Writing – review & editing. EA: Investigation, Methodology, Writing – review & editing. PB: Methodology, Writing – review & editing. MM-R: Methodology, Resources, Writing – review & editing. MT: Investigation, Methodology, Resources, Writing – review & editing. RH: Formal analysis, Investigation, Methodology, Writing – review & editing. MV: Conceptualization, Formal analysis, Investigation, Methodology, Project administration, Resources, Supervision, Writing – original draft, Writing – review & editing.
