# Supplementary material for: Antineoplastic effect of compounds C14 and P8 on TNBC and radioresistant TNBC cells by stabilizing the K-Ras4BG13D/PDE6δ complex
Source: Front Oncol. 2024 Mar 20;14:1341766. doi: 10.3389/fonc.2024.1341766 (PMC10989073; doi:10.3389/fonc.2024.1341766)
Supplement: Supplementary file 1 [file DataSheet_1.docx]

Supplementary Material

# Supplementary Data

Supplementary Material should be uploaded separately on submission. Please include any supplementary data, figures and/or tables.

Supplementary material is not typeset so please ensure that all information is clearly presented, the appropriate caption is included in the file and not in the manuscript, and that the style conforms to the rest of the article.

# Supplementary Figures and Tables

For more information on Supplementary Material and for details on the different file types accepted, please see [here](https://www.frontiersin.org/guidelines/author-guidelines#supplementary-material).

## Supplementary Figures

**Supplementary Figure 1.** The figure legends are required to have the same font as the main text, 12 point normal Times New Roman, single spaced. Please use a single paragraph for each legend and prepare the figures keeping in mind the PDF layout.


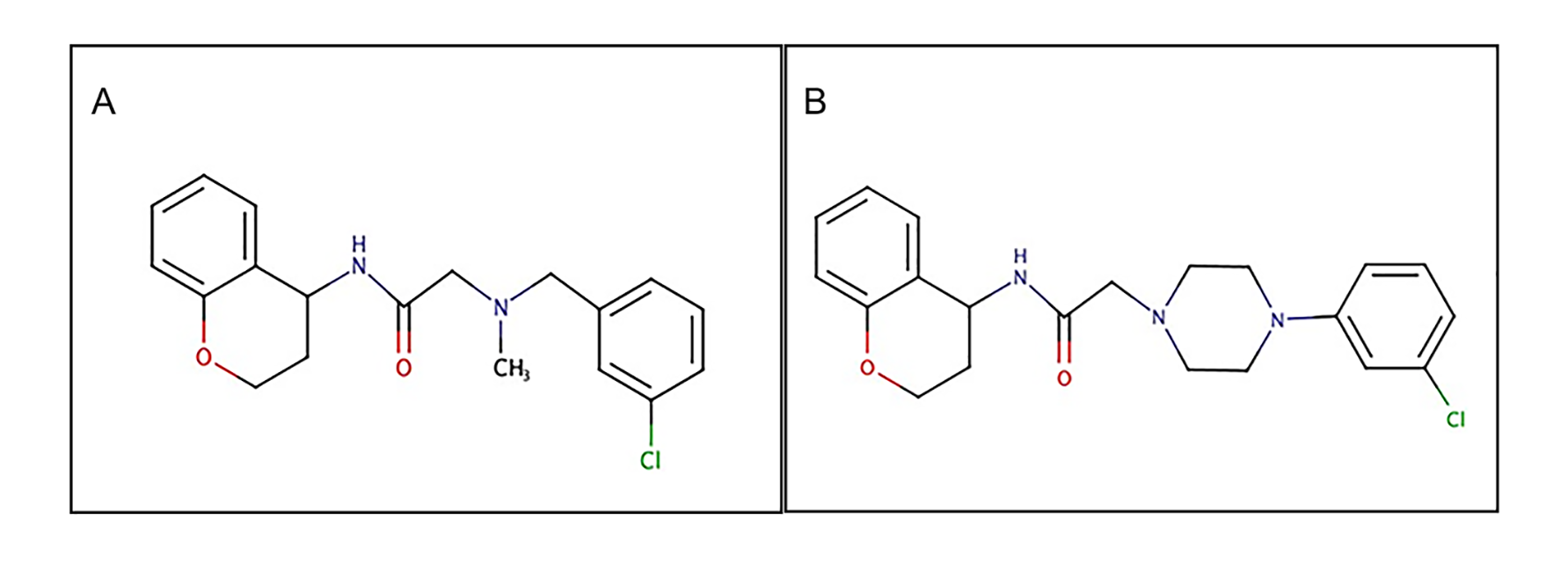


**Supplementary Figure 1.** Compounds identified *in-silico* search. **A**) 2-[(3-chlorophenyl) methyl-methyl-amino]-N- chroman-4-yl-acetamide structure known as C14. This compound's molecular structure includes a chroman moiety linked to an acetamide group along with a chlorophenyl-methyl-amino side chain. Its design suggests potential interactions with cancer-specific targets such as Kras4B or pathways, making it a candidate for inhibiting cancer cell growth or inducing cell death. **B**) 2-[4-(3-chlorophenyl)piperazin-1-yl]-N-[(4R)-chroman-4-yl]acetamide structure known as P8. P8 comprises a chroman structure linked to an acetamide group and a piperazine ring with a chlorophenyl substituent. Its chemical architecture hints at interactions with specific cellular components or processes involved in cancer proliferation or survival, suggesting it as a potential anti-cancer agent.


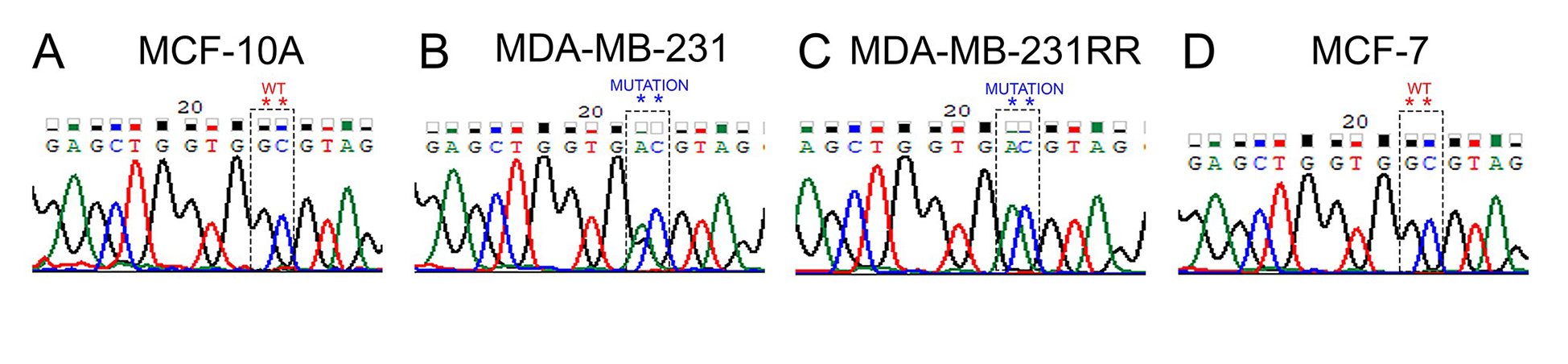


**Supplementary Figure 2.** Chromatograms for KRAS codon 12 and 13, forward primer set. **A**) MCF-10A chromatogram peaks, wild-type control region is shown in dotted rectangle. **B**) MDA-MB-231 chromatogram peaks, mutation is shown in the center of dotted rectangle (G13D). **C**) MDA-MB-231RR chromatogram peaks, mutation is shown in the center of dotted rectangle (G13D). **D**) MCF-7 chromatogram peaks, wild-type region is shown in the center of dotted rectangle.


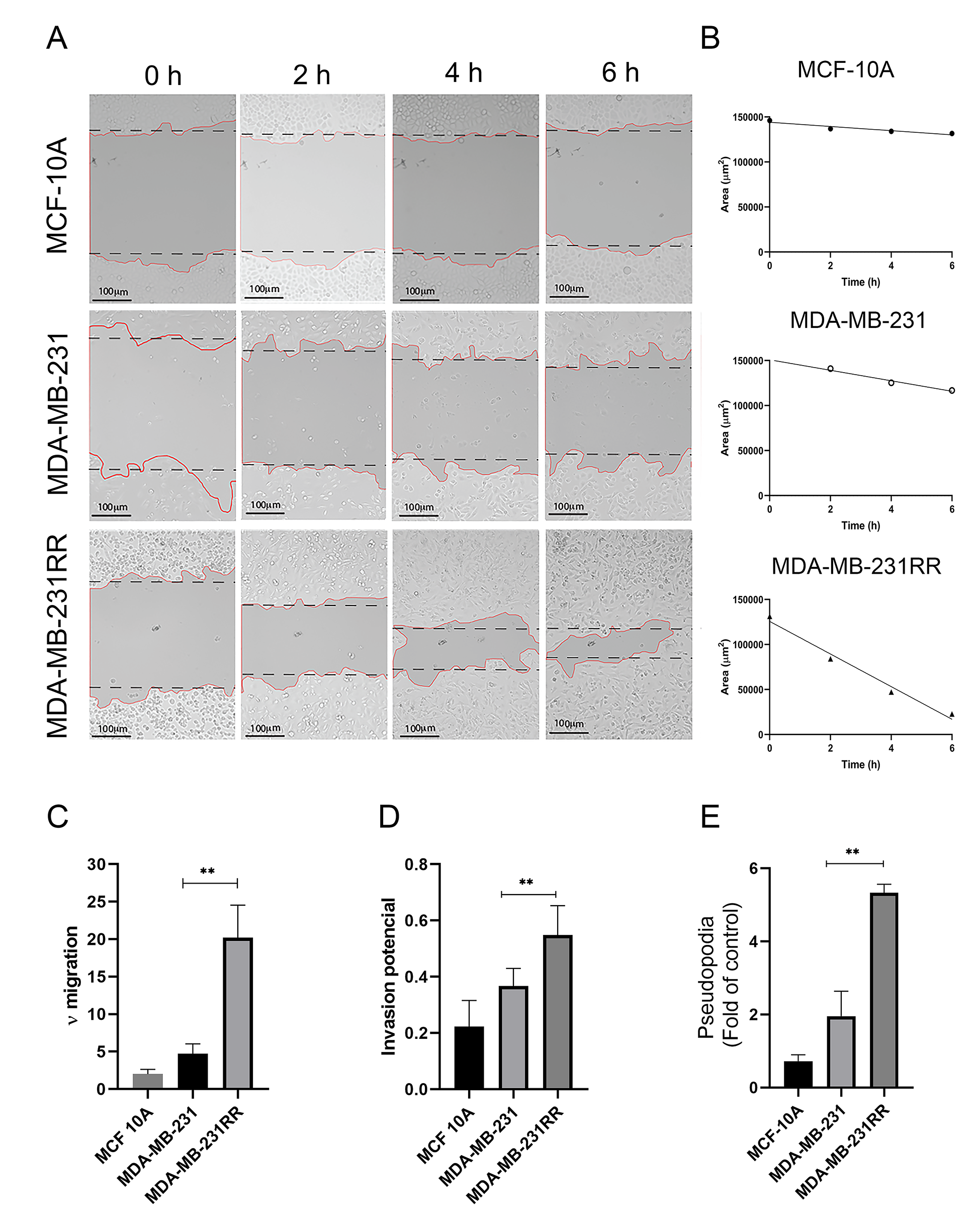


**Supplementary Figure 3.** Evaluation of migration ability, velocity, invasion, and pseudopodia formation of MCF-10A, MDA-MB-231 and MDA-MB-231RR. **A**) Wound-healing assay was carried out at 0, 2, 4 and 6 h. A low cell migration ability of MCF-10A cells is shown in the upper panel. In the middle panel, is shown the cell migration ability of MDA-MB-231. It is higher in comparison with the non-tumoral cell line. Finally, in the lower panels are presented the migration ability of MDA-MB-231RR. In this case, in comparison with the control cell line and its parental cell line, these cells showed the higher migration capability. Scale bar 100 µm. **B**) Quantification of the closure of the "wound" area. Clearly it showed the higher ability of MDA-MB-231RR to migrate. **C**) Velocity of migration of MCF-10A, MDA-MB-231 and MCF-231RR. The highest velocity is disclosed by MDA-MB-231RR cells. **D**) Invasion potential of MCF-10A, MDA-MB-231 and MCF-231RR. The highest invasion potential is observed by MDA-MB-231RR cells. **E**) Quantification of pseudopodia numbers in MCF-10A, MDA-MB-231 and MCF-231RR. The highest amount of pseudopodia is observed in MDA-MB-231RR cells. Rule: 100 µm.


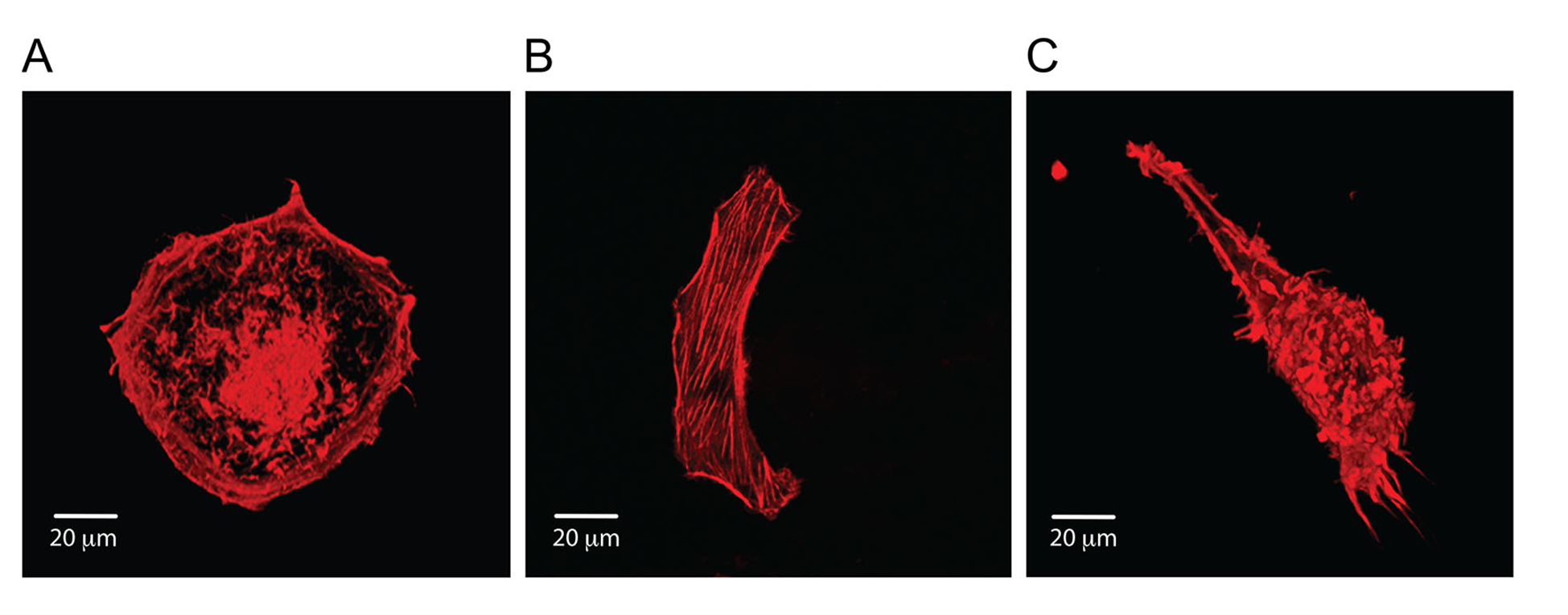


**Supplementary Figure 4.** Evaluation of cellular morphology and expression of malignity markers and lineage markers in the cell lines, MCF-10A, MDA-MB-231 and MDA-MB-231RR. Cell morphology was evaluated by F-actin arrangement labeled in red. **A)** Cell morphology of MCF-10A cells. An epithelial-like morphology with cuboidal epithelial shape and short microvilli rounded the cell body were observed**. B)** Cell morphology of MDA-MB-231 cells. A fibroblast-like morphology is observed with elongated cell shape with a great number of stress fibers across the cell body**. C)** Cell morphology of MDA-MB-231RR cells. A large number of vacuoles or villi on the cell surface were observed. Rule: 20 μM.

**Table Supplementary 1.** Interacting residues between inhibitory compounds with molecular systems

| System | Compound contacts | Forming H-bonds | GDP contacts | Forming H-bonds |
| --- | --- | --- | --- | --- |
| K-Ras4B^WT^/PDE6δ |  |  | **KRas4B**: A11, G12, G13, V14, G15, K16, S17, A18, F28, V29, A59, N116, K117, D119, L120, S145, A146, K147, T148  **HVR2:** M170, G174, K178 | V14, G15, K16, S145, K147, D119, K178 |
| K-Ras4B^G13D^/PDE6δ |  |  | **KRas4B**: G12, D13, V14, G15, K16, S17, F28, N116, K117, D119, L120, S145, A146, K147.  **HVR2**:K167, E168, M170, K175. | G15, K16, K167, E168, K175, E119, S145, A146, K147 |
| K-Ras4B^G13D^/PDE6δ-C14 | **PDE6δ:** A58, W90. L108, I109, E110  **KRas4B**: S17, I21, V29, D33, I36, E37, D38, D57 A59 G60, Y64 **HVR2**: G174, K177, K178, S181, K182.  **GDP** | **KRas4B**: E37  **HVR2**: K177 | **KRas4B**: G12, D13, V14, G15, K16, S17, A18, F28, V29. N116, K117, C118, D119, L120, S145, A146, K147. T148  **HVR2**: M170, K178  **C14** | V14, G15, K16, S17, A18, K117, D119, S145, A146, K147  **HVR2:** K178 |
| K-Ras4B^G13D^/PDEδ-P8 | **PDE6δ:** L108, I109, E110  **KRas4B**: S17, A18, I21, V29, E31, Y32, D33, I36, E37 D38, D57, A59, G60  **HVR2**: M170, D173, G174, K177, K178, S181, K182. | **HVR2**: K177, K182 | **KRas4B**: D13, V14, G15, K16, S17, A18, L19, F28, V29, A79, N116, K117, C118 D119, L120, S145, A146, K147, T148. **HVR2**: M170, K178 | D13, V14, G15, K16, S17, A18, N116, K117, D119, S145, A146 |
